# Functional contextual implementation of an evolutionary, entropy-based, and embodied free energy framework: Utilizing Lagrangian mechanics and evolutionary game theory’s truth vs. fitness test of the veridicality of phenomenological experience

**DOI:** 10.3389/fpsyg.2023.1150743

**Published:** 2023-04-11

**Authors:** Darren J. Edwards

**Affiliations:** Department of Public Health, Swansea University, Swansea, United Kingdom

**Keywords:** Evolutionay game theory, entropy, interoception, graph theory, functional contextualism, predictive coding

## Abstract

The Bayesian approach of cognitive science largely takes the position that evolution drives perception to produce precepts that are veridical. However, some efforts utilizing evolutionary game theory simulations have shown that perception is more likely based on a fitness function, which promotes survival rather than promoting perceptual truth about the environment. Although these findings do not correspond well with the standard Bayesian approach to cognition, they may correspond with a behavioral functional contextual approach that is ontologically neutral (a-ontological). This approach, formalized through a post-Skinnerian account of behaviorism called relational frame theory (RFT), can, in fact, be shown to correspond well with an evolutionary fitness function, whereby contextual functions form that corresponds to a fitness function interface of the world. This fitness interface approach therefore may help provide a mathematical description for a functional contextual interface of phenomenological experience. Furthermore, this more broadly fits with a neurological active inference approach based on the free-energy principle (FEP) and more broadly with Lagrangian mechanics. These assumptions of how fitness beats truth (FBT) and FEP correspond to RFT are then discussed within a broader multidimensional and evolutionary framework called the extended evolutionary meta-model (EEMM) that has emerged out of the functional contextual behavioral science literature to incorporate principles of cognition, neurobiology, behaviorism, and evolution and are discussed in the context of a novel RFT framework called “Neurobiological and Natural Selection Relational Frame Theory” (N-frame). This framework mathematically connects RFT to FBT, FEP, and EEMM within a single framework that expands into dynamic graph networking. This is then discussed for its implications of empirical work at the non-ergodic process-based idiographic level as applied to individual and societal level dynamic modeling and clinical work. This discussion is framed within the context of individuals that are described as evolutionary adaptive and conscious (observer-self) agents that minimize entropy and can promote a prosocial society through group-level values and psychological flexibility.

## Introduction

One well-established assumption within cognitive science is that the cognitive system promotes veridical precepts and that through evolution, natural selection drives increasingly veridical perceptions about the objective world (Marr, 1982, 2010; Palmer, 1999; Geisler and Diehl, 2003; Maloney and Zhang, 2010; Pizlo et al., 2014). There is of course some evidence supporting this claim; for example, the eye, as complex as it is today, was postulated by Darwin (1859) to have emerged from much simpler evolutionary beginnings. Initially, a prototype eye was thought to have evolved through purely stochastic means, which formed and allowed the organism to detect direct light. Given that this light detection would provide the organism a substantial survival advantage over organisms that could not detect light, this adaptation would then be selected and continue to adapt with further evolutionary variation and selection. To support Darwin’s postulate, this prototype eye has subsequently been found in the planarian species Polycelis auricularia and the trochophore planktonic marine larvae (Gehring, 2014). This, according to Darwin (1859), has led to the highly evolved eye we have today that not only detects light but also shape, color, contrast, movement, etc.

In complex organisms, the tapetum lucidum is a layer in the eye of many vertebrates, which sits behind the retina with the sole purpose of reflecting light through the retina, thus allowing more available light to pass through the eye’s photoreceptors. This increases the amount of available light for organisms that have the tapetum lucidum, which allows them to see better in the dark and therefore has obvious survival advantages, particularly if the organism hunts at night or is hunted at night. In fact, organisms that have the tapetum lucidum are typically active at night, such as deer, dogs, cats, horses, and ferrets. Humans, other primates, squirrels, birds, and pigs do not have this structure in their eyes, and this is thought to be because they are diurnal (mainly active during the day; Ollivier et al., 2004). It, therefore, seems that the tapetum lucidum only evolved through selection in animals that directly benefited from it through a direct survival advantage. This gives these organisms greater veridical perception at night than organisms that do not have it (without the use of technology), as they can see light reflect off objects within the world (Bennett & Cuthill, 1994). Importantly, this natural selection advantage in perceiving a veridical world is not isolated to just visual perception; a bat, for instance, can hear ultrasound and uses echolocation to navigate (Jones et al., 2013). Likewise, rats have a higher sense of smell than humans, with a higher number of olfactory receptor neurons and therefore greater veridical olfactory perception (Duchamp-Viret et al., 1999; Keller and Vosshall, 2008). Thus, it seems that all forms of veridical phenomenology are shaped by evolutionary fitness payoffs based on the organism’s context.

However, assuming that any of these perceptions are veridical assumes a naïve realist ontology typical in cognitive psychology, which assumes perceptual mapping is to be exactly veridical, or at least a critical realist ontology, which assumes that at least some aspects of the environment the organism experiences through the senses are based on a “true” veridical reality. Although much of the evolutionary evidence suggests that perception is driven by fitness (contextualized given the organism’s survival or reproductive needs) and not solely by absolute veridical object reality, many cognitive theories have still proposed a naïve realist position (that perception should be veridical and computable through Bayesian decision theory) (Marr, 1982, 2010; Palmer, 1999; Geisler and Diehl, 2003; Pizlo et al., 2014). However, certain mathematical models have also been proposed in the area of evolutionary game theory (Smith and Price, 1973; Smith, 1982; Nowak, 2006) that show and emphasize an entirely non-veridical nature to the perception that evolutionary fitness within these game simulations selects non-veridical perception in nearly all cases (Mark et al., 2010; Prakash et al., 2021).

These findings have broad implications for a philosophy of science in the psychology of the “mind” or “behavior” of an organism. It rationalizes that the “mind” or “behavior” is shaped at its foundation by an evolutionary fitness function rather than specific ontologies such as physicalism, mentalism, or naive realism, ultimately giving rise to an ontologically neutral (a-ontological) stance on these phenomena. This a-ontological position is directly opposed to the naïve (or critical) realist position of cognitive psychology. Instead, it is more aligned to a behavioral position, for example, a post-Skinner contextual behavioral science account based on functional contextualism relational frame theory (RFT; Barnes-Holmes et al., 2001; Blackledge, 2003; Zettle et al., 2016), which is based on behavioral pragmatism and also holds an a-ontological position in explaining how complex human behavior emerges (Barnes-Holmes, 2005; Codd, 2015; Monestes and Villatte, 2015).

Given such an a-ontological alignment, and the fact that recent developments within clinical psychology are suggesting a move away from protocols of syndromes, such as the Diagnostic and Statistical Manual of Mental Disorders (DSM-5; American Psychiatric Association, 2013), and toward a more process-based therapeutic (PBT) approach (Hayes and Hofmann, 2018; Hayes et al., 2019, 2020) that highlights evolution as central (evolutionary variation, selection, retention, and context; Hayes et al., 2020; Hofmann et al., 2022) within the therapeutic practice, it seems appropriate to explore how the evolutionary game theory (Smith and Price, 1973; Smith, 1982; Nowak, 2006) can be applied within the context of this study. This specifically relates to evidence provided through mathematical simulations that show phenological experience (e.g., perception) to be non-veridical and instead act through a perceptual interface based on evolutionary fitness functions (Mark et al., 2010; Hoffman and Prakash, 2014; Hoffman et al., 2015; Prakash et al., 2021).

The evolutionary approach of PBT is defined through the extended evolutionary meta-model (EEMM; Hayes and Hofmann, 2018; Hayes et al., 2019, 2020). This is a multilevel and multidimensional modeling approach that emphasizes data modeling to be driven at the non-ergodic ideographic individual level and not a nomothetic population-driven approach. The EEMM dimensions include affective, cognitive, attentional, self, motivational, and overt behavior and have two levels of analysis that are biopsychological and sociocultural. As the EEMM is an expanded contextual behavioral approach that has traditionally emphasized acceptance and commitment therapy (ACT) at a middle level (Hayes et al., 1999, 2004, 2011) and RFT at the basic level (Barnes-Holmes et al., 2001; Blackledge, 2003; Zettle et al., 2016), an exploration of RFT framing will be performed, as well as the psychobiological level of the EEMM in the form of principles of free energy and the Markovian blanket typically associated with neuroscience (Friston, 2010; Schwartenbeck et al., 2013).

An even deeper contextual analysis is also explored at the level of entropy, related to chaotic dynamical self-adaptive behavior, in which value-directed behavior and other dimensions of EEMM and principles of free energy can lead to a reduction in entropy. This includes principles of Lagrangian mechanics and how this interfaces with perceptual and phenomenological experience based on an evolutionary fitness function. In addition to this, there has been, to date, less attempt to define mathematically a formal framework that directly and explicitly extends the RFT’s relational frames through the EEMM and within an ideographic PBT network approach (an ideographic dynamic network analysis, IDNA), that incorporates evolution, free energy, expected utility, and entropy. The basic outline of one such formal mathematical framework implementation is given here and is called the “Neurobiological and Natural Selection Relational Frame Theory” (N-frame), which emphasizes RFT relational frames projected through the EEMM and free energy that define important mathematical structures called Markov blankets, which are important for defining an unbounded mathematical space for “self” in RFT (i.e., separating observer self from self as content).

This theory and hypothesis paper explores (1) the standard cognitive Bayesian approaches to veridical perception and simulations confirming Hoffman and colleagues’ (Mark et al., 2010; Hoffman and Prakash, 2014; Hoffman et al., 2015; Prakash et al., 2021) suggestion that phenomenological experience such as perception is based on fitness and not veridical Bayesian “truth”; (2) logical and set theory mathematical interpretations of RFT that may be applicable for time series ideographic PBT; this includes how to develop these into graphs of graph theory computationally; (3) problems with rare paradoxical self-referential strange loops that are introduced in formal atomic logical system models and could apply when modeling deictics of RFT “self” in more complex modes; in addition, how Markovian blankets and Lagrangian mechanics maybe one solution to this; and (4) the broader implications of how entropy reduction and embodied self naturally emerge when introducing personal values and Lagrangian mechanics.

## The standard cognitive Bayesian framework for visual perception

A common approach to visual perception in cognitive science (which assumes a naïve realist ontology) is to use a Bayesian approach and assume that perception is veridical (Marr, 1982, 2010; Palmer, 1999; Geisler and Diehl, 2003; Pizlo et al., 2014). According to this approach, given some image *x*_0_, the cognitive-visual system attempts to find the most probable interpretation of the world. To do this, it compares the posterior probability *P* of various interpretations of the world *w* given some image of the environment that is being seen *x*_0_, and this can be denoted as $P\left(w|x_{0}\right)$. When using Bayes’ rule (theorem), the posterior probability *f* of a cognitive interpretation of the world *x* when given some image *w* is given by:


(1) $$P\left(w|x_{0}\right)=\frac{P\left(x_{0}|w\right)\cdot P\left(w\right)}{P\left(x_{0}\right)}$$

The term *P*(*x*_0_) is the posterior probability of some image that does not depend on a cognitive interpretation *w*. Given this Bayes’ theorem interpretation, the posterior probability is determined by the product of two terms: (1) the probability of an interpretation given some image $P\left(x_{0}|w\right)$, and (2) the probability of an interpretation prior to any image presented (prior probability) *P(w)*. These are then divided by *P*(*x*_0_).

This application of Bayes’ rule yields a probability distribution space of possible interpretations called the posterior distribution. It can be interpreted as a naïve realist cognitive approach (or veridical truth strategy) to perception, whereby it gives the highest posterior probability given some image *x*_0_, and it is this strategy that is selected by the perceptual system when processing perceptual “truth.” This strategy consists of a maximum-*a-posteriori* (MAP) that selects the most probable perception given the posterior distribution, according to this Bayes’ theorem approach. Here, a function *f* can be described, whereby a sampling distribution of observations (in this case images) *x* can be made, an unobserved population parameter is given as *w*, and therefore the function f(x|w) is the probability of *x* when the population parameter (or world) is *w*. This is given as w↦f(x|w), and the maximum estimate of *w* is given as ${\overset{⌢}{w}}_{MLE}\left(x\right)=\arg\underset{w}{\max}f\left(x|w\right)$. The Bayes’ rule MAP strategy also depends on a gain or loss function that describes the consequences of making errors. Here, for visual perception, it uses a Dirac-delta loss function, which gives no loss consequence for a correct (or near-correct within a tolerance level) answer and an equal loss consequence for all incorrect answers (or interpretations in the case of visual perception).

## Computational evolutionary perception

In the standard Bayesian approach of perceptual vision, the space *W* plays two distinct roles: (1) it corresponds to the space of objective world states, and (2) it corresponds to the space of perceptual interpretations from which the visual (or more broadly phenomenological) cognitive system must choose. One problem with this standard Bayesian approach for visual perception is that the framework conflates the Bayesian interpretation space with the real world by assuming their structures are homomorphic. However, for the many reasons already given (e.g., the examples of evolution selecting attributes for fitness rather than truth), they are unlikely to be entirely homomorphic.

**Figure 1:**
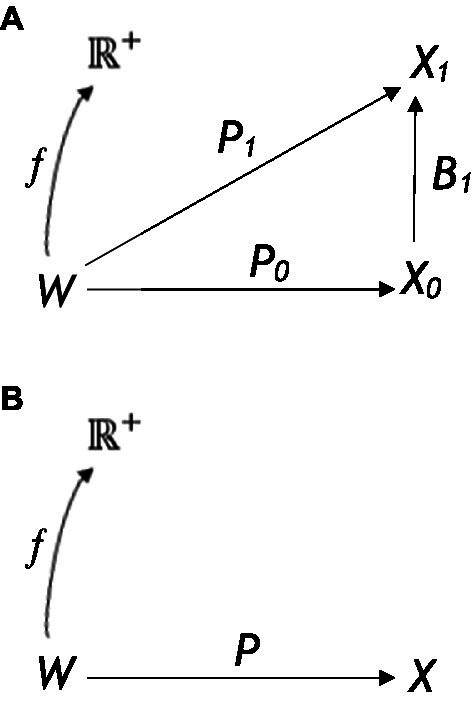
**(A)** The framework of computational evolutionary perception *W* is the observer-independent world, *X*_0_ and *X*_1_ are two perceptual (or representational) spaces, and *P*_0_ and *P*_1_ their respective perceptual channels *f* is a fitness map. In **(B)**, a framework to define the two resource strategies. A fixed perceptual map is assumed P:W→X and a fixed function $f:W\to {\mathbb{R}}^{+}.$ The organism must select a territory associated with the greatest fitness payoff, given a choice of available territories that it identifies through sensory states $x_{1},x_{2},\ldots ,x_{n.}$ [Reprinted with permission from Springer (Prakash et al., 2021)].

An alternative theory that does not assume any homomorphic relation between the Bayesian interpretation space and the objective world (i.e., does not assume perception is veridical) is the framework of computational evolutionary perception (CEP; see Figure 1; Hoffman and Singh, 2012; Dickinson et al., 2013; Hoffman et al., 2015), which instead assumes perception is based on fitness. Here, the probabilistic inference that results in perceptual experience takes place in a separate observer-specific space of perceptual interpretations *X*_1_ that does not need to be homomorphic to *W*.*W* is therefore entirely an observer-independent world; *X*_0_ and *X*_1_ are two perceptual interpretation spaces, while *P*_0_ and *P*_1_ are their respective perceptual channels. The fitness map of this evolutionary approach is denoted as *f*, and perceptual inference is given by the Bayesian posterior map $B_{1}:X_{0}\to X_{1}$ that takes place in *X*_1_ and not *W*. Here, the relation between *W* and *X*_1_ does not need to be homomorphic or even isomorphic, unlike the Bayes’ rule MAP truth strategy.

In the CEP framework, when an image is seen and interpreted as having a 3D shape, it is assumed that this is because of the probabilistic inference in the perceptual space *X*_1_ resulted in such a 3D shape. In this framework, the perceptual interpretation is selected by natural selection, whereby a perceptual interpretation with the highest expected fitness payoff is selected. This means that perceptual interpretations are selected when they lead to a higher expected-fitness payoff (and not those that are most veridical) in the form of more effective interactions with the environment that ultimately promote survival and reproduction. As a simple example of this, if the organism was a hungry lion and the action (behavior) was to eat, then the fitness map *f* may have a high value in a world *w* where meat is highly available (but other forms of food are not). However, if the organism was not meat-eating, such as a rabbit, then *f* may assign a low value to the same state of hunger in the same world *w*. This shows how context-dependent and crucially important functional context is in these types of frameworks (i.e., fitness is based on the specific functional context of the organism, such as meat eating or not, in selecting the correct environment to satisfy the functional cue of hunger). It is for this reason that functional contextualism as an a-ontological approach is highly relevant, particularly when complex human behavioral variation and selection are considered. CEP would, therefore, need to define itself specifically with functional contextualism and integrate itself within a broader EEMM to scale to real-world dynamics with complex human behavior.

As an example of this integration, CEP can describe how given complex higher-dimensional representational structures, such as a 3D representation in *X*_1_, are interpreted rather than a simpler 2D representation of *X*_0_ because it allows a higher expected fitness payoff $P_{1}:W\to X_{1}$ (as depicted in Figure 1A). However, this 3D image does not need to resemble homomorphically or isomorphically the veridical objective world and Hoffman and colleagues provide a fitness beats truth (FBT) mathematical theorem to demonstrate this (Hoffman, 2019; Prakash, 2020; Prakash et al., 2021). Applying CEP within a well-defined functional contextualism context and a broader EEMM can then allow for greater scalability for real-world complex behavior, as functions generally follow similar fitness payoffs rather than being of a veridical fixed “truth” nature. If a person, for instance, drives their behavior in line with fitness that is functionally contextually relevant and sensitive, such as behaving in a way aligned to arbitrary functional context rather than fixed notions of truth about the world, then the payoff is likely to be larger than if they used a simple, fixed veridical strategy. This can be seen, for instance, in the attribution, understanding, and use of concepts when using language embedded within complex conceptual learning histories, and is particularly relevant in artificial intelligence and understanding public health messages (Mulhern et al., 2018; Edwards, 2021; Edwards et al., 2022).

In evolution theory, fitness refers to the probability of transferring genes and associated characteristics from one generation into the next (Darwin, 1859; Smith, 1982). However, different decisions and behaviors (actions) *a* of an organism or population can also have a fitness value, and this can be represented as a global fitness function f(w,o,s,a) that depends on the state *w* of the world *W* in which the behavior takes place, the organism type *o* that is making the behavior (e.g., human, lion, and dog) that provides context to the behavioral states, and the state *s* of the organism (hungry, thirsty, fearful, etc.). Fitness functions between organisms can vary greatly, sharing less correlation with one another (hence the importance of functional contextualism, as each organism will have different functional needs). For any particular organism, the complexity of the fitness function grows rapidly as the number of possible states and actions increases.

From the perspective of evolutionary game theory (Smith and Price, 1973; Smith, 1982; Nowak, 2006), the behavior of different organism types is competing for fitness points as they interact in some shared environment *W*. In an evolutionary-based competitive game, natural selection favors the precepts, decisions, and behaviors of these organisms that yield higher fitness points. In a very simple game, whereby all the organisms are of the same type *o*, have the same state *s*, and have a single available behavior (or action) *a*, this can be modeled as a specific fitness function. The function can be described as a nonnegative real-valued function defined on world *W*. The function can be denoted as f:W→[0,∞) and means that only the state *w* of the world *W* (and not the state *s*, organism *o*, or behavior *a*) can vary between a value of zero and a value below infinity.

Using this approach, the fitness of different perceptual and behavioral decision strategies is compared through an evolutionary resource game (Mark et al., 2010; Hoffman et al., 2013). In a typical game, the game consists of two organisms, utilizing different strategies and competing over territory, with a limited number of resources. In the first instance, available territories are observed by the first “player,” and the player chooses the territory that they estimate to be the optimal one (the highest expected fitness payoff), then receives a fitness payoff for that chosen territory. The second “player” then does the same with the remaining territories available and receives a fitness payoff for that chosen territory. Then the two players take turns in choosing the remaining territories, receiving a fitness payoff for each chosen territory, and both trying to maximize their fitness payoffs for the territories they choose.

Here, the relevant world attribute *w* is the number of resources in some given territory, and *W* is depicted as the world of different quantities of some resource in different territories. A perceptual map can then be considered P:W→X, whereby *X* is a set of perceptual states (for instance, red, yellow, green, and blue) in the world state *W*, and this, for example, could equal some arbitrary value such as one to a hundred different world states, i.e., from *W* = [1,100], while X={R,Y,G,B}. In this approach, perceptual map *P*, therefore, selects the best perceptual element state of the set relevant to the strategy being employed.

A fitness function of the world W,f:W→[0,∞) assigns a nonnegative fitness value to each resource quantity for each territory between zero and a number below infinity. Some functions can be monotonic (whereby as resources keep increasing, fitness also increases), while others can be nonmonotonic (even if resources keep increasing, fitness peaks at a certain number of resources). Nonmonotonic fitness functions are more likely in the natural world as having too many resources may eventually not serve any advanced (e.g., too many food resources have no advantage if excess cannot be stored). As energy resources are limited, the organism is likely to balance the effort made in obtaining resources and the need to obtain resources.

One important assumption of this evolutionary approach is that fitness does not need to correlate in any way with a fixed, veridical “objective truth” about the world. This is because, though fitness depends on the world the organism lives in, it also depends on the organism, and crucially the organism’s perceptual states, as well as the organism’s behavioral action class being considered. In other words, even if the world remains the same, if the action class, perceptual state (or functional context), or even the organism changes, then very different fitness values can be formed. Thus, fitness is entirely functionally context-dependent based on the functional context of the organism and not based on fixed veridical objective truth.

In evolutionary game theory, the organism’s behavior depends (see Figure 1B) on the following three elements: (1) the fitness function as defined by its states, action class, etc.; (2) its prior probabilities of world states; and (3) its perceptual map from a world state *via* sensory states (i.e., resources in territories *w* estimated *via* different sensory states *x*). The organism observes a number of territories in any given trial (or turn in the game), and these are available through its sensory states, $x_{1},x_{2},\ldots ,x_{n}$. The goal of the organism (in this example) is to select a territory associated with the greatest fitness payoff (i.e., the greatest resource fitness payoff). In this case, two possible strategies are competing against one another: a perceptual truth strategy consistent with a naïve realist cognitive approach or a fitness-only strategy that is more consistent with a functional contextual approach.

For the standard perceptual truth strategy typical of a cognitive science approach (that assumes evolution is driving veridically true perceptions), the organism estimates the world state (territory resources in this example) for each of the *n* sensory states $x_{1},x_{2},\ldots ,x_{n}$. Objective “truth” is given by the Bayesian MAP estimate that gives the world state the highest probability of being the true one, given the sensory state. The fitness values of the *n* “true” world states are then compared, and finally, a choice of which territory to select is made. This choice is ultimately based on the sensory states *x_*i*_* that yield the highest fitness values. Crucially, the choice is mediated through the MAP estimate of the world that it considers to be absolutely true and ignores any information about other possible states of the world other than the one being selected as true.

In the fitness-only strategy, the organism does not attempt to estimate the “true” world state for each sensory state. Instead, the expected fitness payoff that results from each possible choice based on sensory states *x_*i*_* is utilized. Given the possible world states, there is a posterior probability distribution for each given sensory state *x_*i*_* and a fitness value for each given world state. These fitness values are then weighted through the posterior probability distribution. This is done so that the expected fitness values for a given choice *x_*i*_* can be computed, and the highest expected choice fitness is then ultimately chosen.

In an example of an actual evolutionary game between two strategies, *A* and *B,* the payoff matrix is illustrated in Table 1A. Payoffs are denoted by *a*, *b*, *c*, and *d* for a row player against a column player, for example, *b* is the payoff to *A* when *A* plays *B*. Fitness payoff *a* is defined as “fitness-only when playing against fitness only,” fitness payoff *b* is defined as “fitness-only when playing against truth,” fitness payoff *c* is defined as “truth when playing against fitness-only,” and fitness payoff *d* is defined as “truth when playing against truth.” There are three main theorems from evolutionary game theory that are relevant to such an analysis, which are true through mathematical proofs (Nowak, 2006):

**Table 1:** A payoff matrix in evolutionary game theory for two strategies, A and B.

A		Against *A*		Against *B*
*A* plays		*a*		*b*
*B* plays		*c*		*d*
B	Likelihood of *w_j_* given *x*_1_, $P\left(x_{1}|w_{j}\right)$	Likelihood of *w_j_* given *x*_2_, $P\left(x_{2}|w_{j}\right)$	Prior $P\left(w_{j}\right)$	Fitness $f\left(w_{j}\right)$
*w*_1_	1/4	3/4	1/6	19
*w*_2_	3/4	1/4	3/6	5
*w*_3_	1/4	3/4	3/6	4
C	Likelihood of *w_j_* given *x*_1_, $P\left(x_{1}|w_{j}\right)$	Likelihood of *w_j_* given *x*_2_, $P\left(x_{2}|w_{j}\right)$	Prior $P\left(w_{j}\right)$	Fitness $f\left(w_{j}\right)$
*w*_1_	3/4	1/4	1/5	6
*w*_2_	1/4	3/4	3/5	5
*w*_3_	3/4	1/4	3/5	21

Fitness payoff a is defined as “Fitness-Only when playing against Fitness-Only,” fitness payoff b is defined as “Fitness-Only when playing against Truth,” fitness payoff c is defined as “Truth when playing against Fitness-Only,” and fitness payoff d is defined as “Truth when playing against Truth.” B demonstrates the fitness beats truth in the evolution of perception values. C demonstrates the fitness beats truth in the evolution of perception after a major environmental change.

**Theorem 1.** In a game with a finite population of two types of players, *A* and *B*, if *b* > *c*, *a* > *c*, and *b* > *d*, for all *N*, $h_{i}>0\forall i$ and $\rho_{AB}>\frac{1}{N}>\rho_{BA}$, then selection favors *A*.

**Theorem 2.** In a game with a large finite population of two types of players, *A* and *B,* and with weak selection, $\left(a-c\right)+2\left(b-a\right)>\frac{2\left(a-c\right)-\left(b-d\right)}{N}\;$implies that $\rho_{BA}>\frac{1}{N}$. Therefore, if *a* > *c* and *b* > *d* for large enough *N*, then selection favors *A*.

**Theorem 3.** For all possible fitness functions and *a priori* measures, the probability that the fitness-only strategy dominates the truth strategy is at least (|X|−3)/(|X|−1), where |X| is the size of the perceptual space.For a simple example where there are three world states, $W=\left\{w_{1},w_{2},w_{3}\right\}$ and two sensory state stimulations $X=\left\{x_{1},x_{2}\right\}$, there is a likelihood value for each sensory stimulation given a world state, and this is given by $P\left(x_{1}|w\right)$ (see Supplementary material 1, which uses the example in Table 1B). To summarize, from this “truth” observer strategy calculation, for state *x*_1_, the truth (i.e., the maximum-a-posterior) estimate is *w*_2_ and for state *x*_2_, the truth estimate is *w*_3_. The truth observer is thus essentially given a choice between selecting the resource *w*_2_ or *w*_3_ (when given sensory state stimulations *x*_1_ and *x*_2_) that contain food, and whereby *w*_2_ has higher quality food and thus higher fitness value than *w*_3_. Given the natural selection, the food (world *w*) with the highest fitness is then selected, and in this case, it will therefore prefer *w*_2_ as fitness value $f\left(w_{2}\right)=5$ is greater than fitness value $f\left(w_{3}\right)=4$. When using this fitness payoff, and then when offered a choice between *x*_1_ and *x*_2_, the truth observer will choose *x*_1_ (corresponding to *w*_2_) with an expected fitness utility of 5.85 (as $f\left(w_{2}\right)$ yielded the greatest direct payoff in the previous steps 3 and 4).

In contrast to this, a “fitness-only” observer is not attempting to calculate the veridical truth about the environment through a Bayesian process. Instead, the “fitness-only” observer is only concerned with which sensory experience yields a higher expected fitness (i.e., maximizing expected fitness). Thus, for a fitness-only observer, step 4 gives these fitness values for both *x*_1_ and *x*_2_. As the expected fitness utility for *x*_1_ is 5.85 and the expected fitness utility for *x*_2_ is 6.17, given a choice between sensory states *x*_1_ and *x*_2_, the organism using a fitness-only observer will choose the higher value of 6.17, and thus chooses the state *x*_2_. From this analysis, it is therefore clear that the truth observer minimizes expected fitness while the fitness-only observer maximizes expected fitness. In this type of evolutionary game theory, the fitness-only observers will therefore drive the population of truth observers into extinction. Hence, perception, given evolutionary game theory, is likely to be based on fitness and not truth. For other examples that account for more complex situations such as sudden environmental change, please refer to Supplementary material 2, which uses the example in Table 1C.

However, despite the ability to account for environmental change, the games employed are very simple. When dealing with real-world scenarios, such as individuals with mental health disorders, modeling these types of evolutionary game theory simulations is much more challenging. In these types of scenarios, their sensory states are likely to be distorted, such as experiencing negative mood states and a lack of motivation, which all would need to be modeled in an evolutionary game in a real-world context. Given the fact that these games within evolutionary game theory are based on functional contextualism, such that fitness in these games is based on a combination of the organism’s sensory state *x* (such as hunger, which is the function of that organism driving its behavior at time point *t*_1_) and the context of a world *w*, i.e., whether the world has what the organism needs to satisfy its functional state (if it does, then that world *w* has a high fitness value *f* for the organism’s current functional state *x* that drives its behavior), then these games may benefit from the application of a formal operational definition for functional contextualism, and within a broader EEMM so that it could potentially be usefully applied to clinical settings and PBT research.

**Figure 2:**
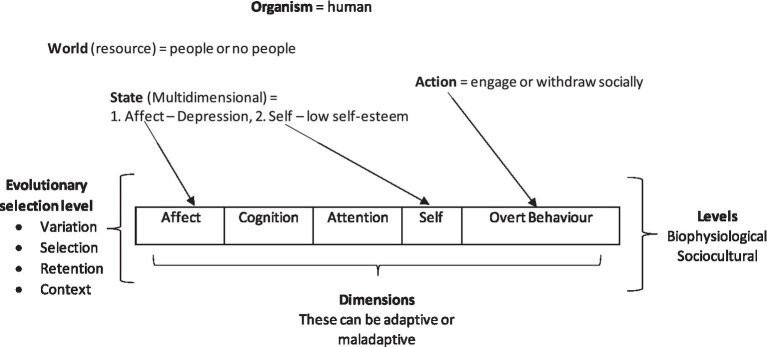
An illustration of how the evolutionary game theory constructs fit and are enhanced by EEMM in “evolutionary game simulations” that involve complex mental health outcomes.

Given this assumption, greater modeling efforts that increase the dimensionality of the interface could strengthen the interface’s fitness. For example, the EEMM, an extension of the functional contextual approach, suggests that there are six dimensions (affective, cognitive, attentional, self, motivational, and over-behavior) at two levels (biopsychological and sociocultural), as the approach is evolutionary meta-model; these represent appropriate dimensions for adaptive organism behavior. Figure 2 shows a simple example of how a human with the states of depression and low self-esteem connects to two dimensions of the EEMM. These dimensions that were identified after an examination of 55,000 studies, whereby 72 measures that had successfully mediated intervention outcomes were replicated, were extracted and summarized into the five dimensions and two levels of the EEMM (Hayes et al., 2022; as shown in Figure 2).

These dimensions may provide a much richer context for modeling relevant clinical processes that could be exploited in such games. For example, the affective dimension relates to emotional states; thus, a game could be designed that defines a sensory state *x* as having some negative effect and some resource in a world *w* that brings about positive affect. Fitness *f* would then be determined based on wellbeing, whereby worlds that bring about greater positive affect are fitter than those that do not. Similarly, the cognitive dimension relates to problem-solving, hope, and attitude; attentional dimensions relate to mindfulness and attentional control; motivation relates to values commitment; and overt behavior relates to goal-striving persistence. These dimensions can therefore be applied as states *x*, and resources within worlds *w* are chosen based on the dimensions of EEMM. Selection level of variation, selection, retention, and context are of course relevant in these games as indicated by EEMM, whereby if the context changes, e.g., the world changes due to environmental shift and can no longer provide a positive effect, then it is no longer retained, and selection begins again.

**Figure 3:**
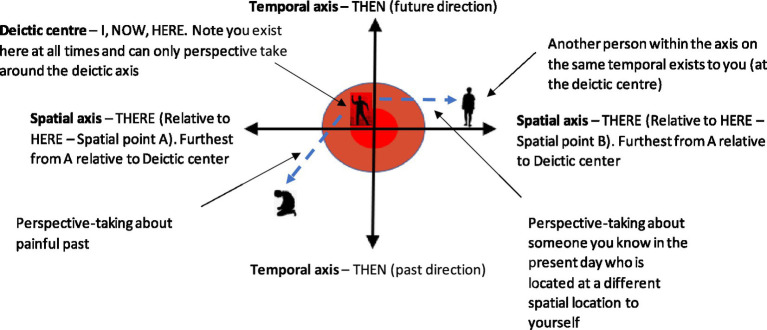
The deictic axis—a visual representation of an individual who relationally frames themselves in the present moment while mindfully observing their painful past. This would be expressed as “I” in the “HERE” and “NOW” as “I” perspective-take about my painful past in the “THERE” and “THEN.”

In one concrete example, the self-dimension is selected in the Figure 2 example, and this self-dimension can be further illustrated by the deictic axis (Figure 3). This breaks this complex dimension of self into its subcomponents of perspective-taking relational frames from the functional contextual RFT model, in the form of the interpersonal (I vs. YOU), the temporal (NOW vs. THEN), and spatial (HERE vs. THERE). In an evolutionary game, this may provide rich context that can define which sensory states are accessed under what context (such as thinking about the future or past) and can potentially increase fitness payoff as a result of this context. For example, if resources in a game are limited, then having perspective about the other player’s state space may help the player avoid direct territorial conflict and select the most optimal world. For example, prosocial behavior may be explained as individuals tapping into collective perspective-taking states about the others’ collective needs, and hence shared goals emerge, which increase fitness for the group as well as the individual, hence encouraging prosocial selection of behavior (Atkins et al., 2019). Crucially, maximizing fitness payoffs of a much higher magnitude would not be possible for the individual to obtain alone.

One current problem is that there has been less effort to mathematically define a functional contextual RFT model within the context of EEMM and evolutionary game simulations in a way that could be visualized within graphs of graph theory and be usefully applied in PBT studies. Within graphs, these should represent functional analytic variables and more broadly patterns of arbitrary applicable responding, that have varying influence over a broader relational network. This will then have scope to connect RFT to the dimensions of EEMM and within the context of evolutionary games. One such approach is to define in detail how relational frames using logic and set theory can be encoded into graphs.

## Set theory and formal relational frame logical axioms represented within community network graphs

Within the functional contextual relational frame theory (RFT), there are several patterns of arbitrary applicable responding, or types of relational frames, which include frames of coordination (stimuli *x* is the same as stimuli *y*); frames of distinction (stimuli *x* is not the same stimuli *y*); frames of causation (if *x* occurs, then *y* will follow); frames of comparison (e.g., *x* is bigger than *y*); frames of opposition (e.g., left is the opposite of right); frames of hierarchy (e.g., Alsatian is a type of dog); and deictic relational frames (perspective-taking), which involve some self-reference of the impersonal relational frame (I vs. YOU), the spatial relational frame (HERE vs. THERE), and the temporal relational frames (NOW vs. THEN).

These relational frames can be formalized in mathematics through the derivation or deduction of properties or propositions (formalized through logic) with respect to objects or elements belonging to a set (general properties of elements and sets can then be formalized through graph theory), and therefore formalized within the set theory and mathematical logic (Pereyra, 2020). There is a natural relationship between set theory and logic; for example, if *A* is a set, then proposition P(x)=x∈A is a logical formula of that set, which states that a proposition holds for some value of *x* in a domain (or set) associated with *x*. The proposition is therefore true for elements of set *A* and false for elements outside of set *A*.

Within mathematical logic, there are subbranches of propositional algebra and predicate logic. A proposition is any statement that can be assigned a unique value of either true or false and cannot be both true and false (the law of excluded middle). As an example of how to apply simple logic (without set theory) to relational frames, A⇔B denotes that *A* and *B* are equivalent, whereby ⇔ is the mathematical operator for equivalence. In RFT, deriving stimulus relations is a special generalized form of relational responding (Gross and Fox, 2009), and the logic symbol ⇒ can be used to symbolize this as it is the operator for “implies” (or “derives”). Thus, if *A* is bigger than *B*, then it can be derived that *B* must be smaller than *A*, and this can be expressed as *A* is bigger than *B* ⇒ *B* is smaller than *A*.

This can be taken a step further, by applying equivalence to relational frames when applied to set theory. There are three classes for this, namely, reflectivity, symmetry, and transitivity. In set theory, if *A* is equivalent to B(A⇔B), then some element *x* of set *A* (x∈A) must also be equivalent in set B(x∈B), so that x∈A⇔x∈B. A very simple equivalence relation can be given by ~, and this is the relation between elements of sets or two sets that hold three conditions: (1) reflexivity a~a; (2) symmetry a~b⇔b~a; (3) transitivity is expressed as (a~b)∧(b~c)⇒(a~c) whereby if *a* and *b* are equivalent, and *b* and *c* are equivalent, then, therefore, *a* and *c* must be equivalent. In RFT, transitivity is called combinatorial entailment. The equivalence relation ~ also applies to sets, such that set *A* equivalence can be used to partition the set into a series of subsets of *A* that are equivalent to each other. The equivalent elements contained in these subsets are equivalent classes. According to categorization theory and RFT work, these shared relations of equivalence do not need to be of physical shape or size; they could, for instance, depend on some contextually dependent function such as the concept of cutlery sharing the equivalent function “to eat with” within an equivalence class subset (such as fork, knife, and spoon).

Elements here do not need to relate to other elements with just equivalence; they can also express other relations, such as “larger than” and “faster than,” through propositional algebra. Propositional algebra is a subbranch of mathematical logic relating to propositions and logical operators. Any statement that can be assigned a unique logical value of “true” (*T*) or “false” (*F*) can be described as a proposition. Logical operators can be used to define a new proposition *S* from one or more given propositions, such as propositions *A* and *B*. In this type of situation, the logical value of the new proposition *S* depends on the logical values of the propositions *A* and *B*. All of the possible combinations of logical values can be presented in a truth table for the propositions *A* and *B* that indicates the corresponding logical value for *S* for each combination. A truth table, therefore, determines the value of the logical operator. In a simple example of this, the BOTH-FALSE operator ⊗ can be used between two propositions, e.g., *A* and *B*, such as *A* ⊗ *B* and is only true (*T*) only if both *A* and *B* are both false (*F*).

The main formal logical operators are NOT, OR, AND, IMPLIES, and EQUIVALENT. Operators such as the NOT operator ¬ can be defined in the following way: ¬A≡A⊗A, whereby ≡ means something is either identical or similar to another element or set but not necessarily equal to it. The relation frame of opposition can be defined as $A=\left(-B\right)$, whereby *A* equals the opposite of itself, expressed as $A=-\left(-A\right)$, or in another example, the color black is equivalent to the opposite of white, expressed as $Black\equiv -\left(White\right)$. Going beyond simple logic to define a relation of mutual entailment in logic and set theory, whereby including sets is important when concepts are considered categories (or sets of elements) of things in the world, mutual entailment expresses a relation between two variables or statements in which one statement or variable logically implies the other and vice versa. This means that if one of these variables is false, or assigns some operator, it also applies to the other mutually entailed variable. To express a relational frame of mutual entailment of two variables *A* and *B*, this can be expressed in set theory as A⊆B and B⊆A. This means that *A* is a subset of *B* and *B* is a subset of *A*, or more generally, all the elements in *A* also belong to *B* and all the elements in *B* also belong to *A*. For example, the mutual entailment between the spoken word “snake” and the concept of an actual snake within the real world, can be represented in set theory as follows ({} represent the category or set of some elements contained inside):*Verbal word "snake" = {x*|*x is the verbal word "snake"}, Actual snake = {y*|*y is an actual snake}, Verbal word "snake"* ⊆ *Actual snake, Actual snake* ⊆ *Verbal word "snake"*.

Combinatorial entailment can be given in set theory, as in the following example: If *a* is bigger than *b* and *b* is bigger than *c*, *a* is, therefore, bigger than *c*. This can be expressed using logic and set theory as follows, with these steps: (1) Define the sets *A*, *B*, and *C*, where *A* represents the set of all elements that are bigger than element *b*, *B* represents the set of all elements that are bigger than *c*, and *C* represents the set of all elements that are smaller than *b*; (2) define the logical statement “*a* is bigger than *b* and *b* is bigger than *c*” and this is denoted as (a∈A)∧(b∈B); (3) The definitions of sets *A*, *B*, and *C*, can then be used to rewrite the logical statements as follows: (a∈{x|x>b})∧(b∈{y|y>c}), which can be read as “*a* is an element of the set of all elements that are bigger than *b*, and *b* is an element of the set of all elements that are bigger than *c*.” To simplify the definition, the set-theoretic notation can be used to express the statement *a* is bigger than *b* and *b* is bigger than *c*, as follows: a>b∧b>c. Finally, to express the combinatorial entailment, this requires simply adding the implies operator ⇒a>c, so that a>b∧b>c⇒a>c.

For the relation of opposition, if there are two variables, *A* and *B*, this can be expressed in set theory as follows:


A={x|xisthepropertyorcharactisticthatcanbetrueorfalse}

B={y|yistheoppositepropertyorcharacteristicofx}

This can then be represented as A⊥B, which means that *A* and *B* have opposite properties, such that *A* could be true and *B* could be false, or vice versa. This can also be represented as A∩B=Ø, which means that *A* and *B* have no elements in common, i.e., there are no values that belong to both *A* and *B* at the same time. For example, this opposite relational frame can be used between two variables “being stronger” and “being weaker” as follows:

Strong={x|xisapersonwhoisstrong}, Weak={y|yisapersonwhoisweak}, Strong⊥Weak, Strong∩Weak=Ø. (For a more complete list of relational frames including the transformation of function (ToF), refer to Supplementary material 3).

**Figure 4:**
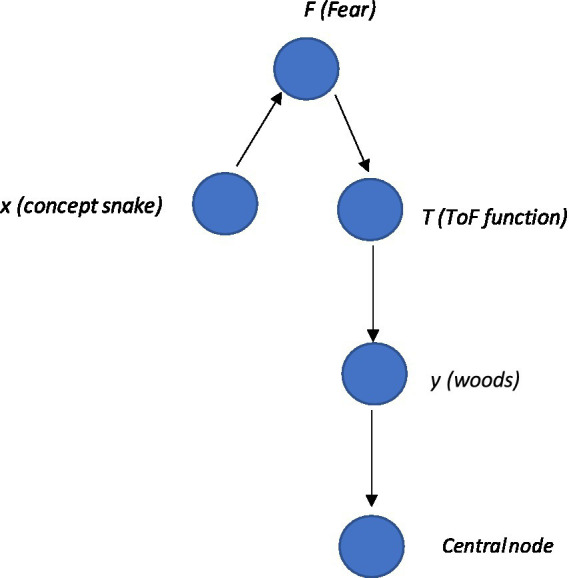
The basic schematic of the graph, whereby snake node *x* is framed with fear node *F* (illustrating the function of the snake as fear), and connecting with the computational function node *T* that expresses the ToF from fear *F* of snake *x* to local woods *y*. In addition, one central node receives input from the whole community, including a ToF between two nodes.

These can be represented visually in a very simple network graph with the following steps: (1) first define a set of nodes where each node represents an element in the community (e.g., snake or woods—see Supplementary material 4 Python code as an example^1^). Nodes can also represent functions such as ToF and relations in some cases; (2) then define a set of edges, where each edge represents a relational frame between two nodes. For example, the hierarchical frame of a snake in the woods can be expressed with edges that represent the hierarchical relationship between concepts represented by the nodes. The edges can be represented visually by directed lines pointing from a node representing a parent to a node representing a child (i.e., the child is a sub-element of the parent). Edges can appear differently, representing different frames such as shades, colors, and labels. This can be expressed mathematically, whereby E is some relationship and n is some node, which can be deonted as $E=\left\{\left(n_{1},n_{2}\right)|n_{1}\;is\;a\;parentof\;n_{2}\right\}$; (3) then use graph theory techniques such as traversal algorithms or centrality measures to explore the relational frames within the network community and identify patterns and trends. This can be made more specific by including a node function, *T*, that transfers (ToF) the set of things you are afraid of, *F*, to the set of woods that you are not afraid of, *W*. In terms of the code, one way of doing this is to create a function (see Supplementary material 5) that simply transfers “woods” directly into the set of things the individual is afraid of. Notably, within a subgraph, one community central node can receive inputs from the community and produce output that comprises the total weight within the community. (Refer to Figure 4 as an illustration of this with the ToF function node *T* included).

**Figure 5:**
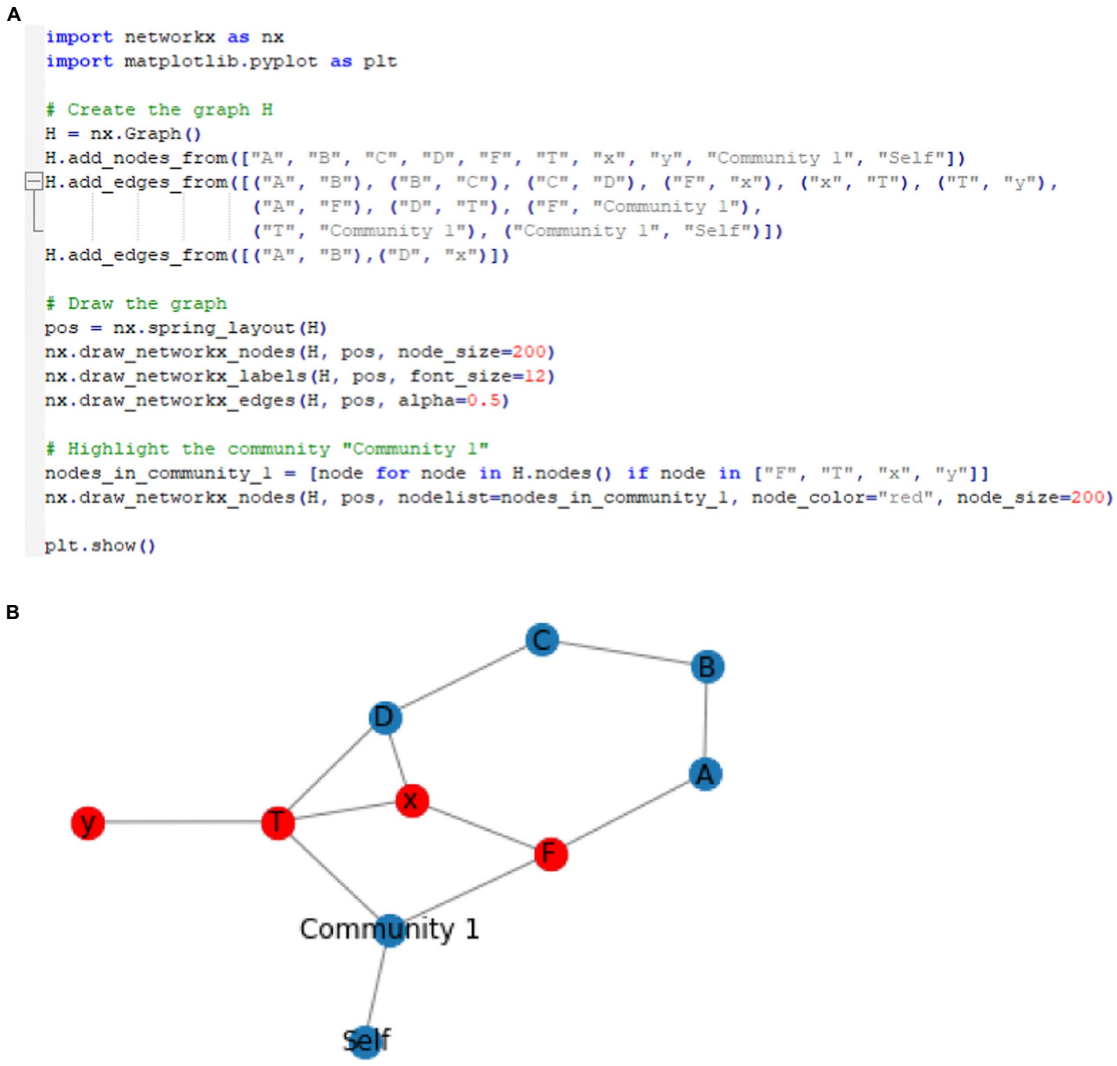
**(A)** Example of Python code for specifying two communities, and the resultant community network where the ToF community (function *T* node) within a broader relational network can be visualized within the graph. **(B)** Visual areas of interest within the network may be an area that a PBT therapist would consider targeting to undermine its negative impact across the network. It also shows in the resultant visualized network whether the ToF function *T* transfers fear *F* of snake *x* to local woods *y*.

In Python programming language, the NetworkX library can be used to create a graph. The community can be analyzed through nx.pagerank and nx.shortestpage functions. This can be represented as a community graph (i.e., a subset of the larger graph that includes sets that relate to one another closely, such as a network that defines the self) within a larger network graph (of multiple communities that relate networks of relating relations). Here, a graph of nodes represents the sets *F* and *T* as well as elements *x* and *y*. The graph could be defined as G=(V,E),V={F,T,x,y}, and E={(F,x),x,T),(T,y)}. Node *F* again represents the set of all things that you are afraid of, and node *T* represents the function that frames snakes with local woods. This is different from the representation that simply associates simply associates snakes with “woods” directly. Instead, this function is a more accurate way to present the variables as the networks increase in size and complexity. Node *x* represents an element in set *F* (i.e., an instance or a stimulus that you are afraid of, such as “snake”), and node *y* represents an element in the set for the function *T*(*F*).Here, the edges (*F, x*) and (*T, y*) represent the membership of *x* in *F* and *y* in *T*(*F*),respectively, while the edge (*x, T*) represents the mapping of framing *x* with *y* through the function *T*. From this, the graph (or community network subgraph), for example, could be defined as G=({"F","T",x",y},{("F","x")("x","T"),("T","y")}). This is illustrated visually within a graph and Python code as depicted in Figure 5. Here, it is also important to note that certain nodes of interest within a community can be highlighted within Python, such as the ToF. This represents an important area of interest when doing a PBT analysis, as targeting the ToF function within the network with, for example, a mindfulness intervention, may undermine the function’s transfer strength and have a significant positive cascading effect across the network. As can be seen, ToF projects to the community node, and this has a direct causal effect on the community of “self,” potentially increasing generalized fear and ultimately increasing the possibility for increased low self-esteem.

It is also useful to note that graph *G* can be represented as a single community within a larger graph of other communities, i.e., representing relational network subgroups (or communities) relating to other relational network subgroups, such as the ToF within the community for “self” (see Supplementary material 6 for details).

## Implementing set theory and logic of RFT into structural equation modeling and graphs

There has been some recent concern over the ergodic approach that provided much of the rationale for nomothetic population-level statistical approaches (Hayes and Hofmann, 2018; Hayes et al., 2019, 2020; Ciarrochi et al., 2022). (For a full discussion, refer to Supplementary material 7). It is for these reasons that an individual idiographic time series approach to an RFT implementation in SEM (an ideographically driven SEM) is explored as opposed to less individually sensitive nomothetic approaches, with the main focus on a highly sensitive ideographic, autoregressive timeseries approach that meets the criteria for assessing intra-subject variability in participants at the individual level.

Pearl’s structural causal modeling (Pearl, 2000) has been successfully applied to causal reasoning (action and change) in artificial intelligence (AI; Pearl, 1988), statistics, economics (Pearl, 2009), social sciences (Pearl, 2000), and even as Bayesian networks represented as propositional reasoning in cognition (Pearl, 2011). This is to extend the ability of classical logic to describe properties within RFT (as described in the previous section) and to allow for a more general mathematical description of causation, which will eventually allow for graph theory modeling and therefore be applicable in a clinical and PBT context.

Pearl (2000) suggests that a causal model is a triple M=〈U,V,F〉 whereby *U* is a set of background exogenous variables, *V* is a set of endogenous variables $\left\{V_{1},V_{2},\ldots ,V_{3}\right\}$ that are determined by variables in the union U∪V. *F* is a set of functions $\left\{f_{1},f_{2},\ldots ,f_{n}\right\}$ where $f_{i}$ is a mapping from $U\cup \left(V\backslash V_{i}\right)$ to $V_{i}$ and a mapping from *U* to *V* is formed from the entire set of *F*. *F* can be represented as set of equations:


(2) $$v_{i}=f_{i}\left(pa_{i},u_{i}\right)\;i=1,\ldots ,n$$

Whereby, *pa_*i*_* is any unique minimal set of variables in $V\backslash \left\{V_{i}\right\}$ (parent variables) that is sufficient for representing *f_*i*_*, and for $U_{i}\subseteq U$. According to Pearl, particular “causal worlds” are determined by instances of U=u exogenous variables. Structural equations encode causal information, whereby anything left of the equal sign is the effect and anything right of the equal sign is the cause. Therefore, here the equal sign conveys the asymmetrical relation “is determined by.”

In the theory of Pearl’s (2012), structural equations are intended to be modular, whereby one equation can be modified without changing the others. This allows the determination of three types of queries that can be asked with respect to a causal model: (1) predictions, i.e., will *X* happen in the event of *Y*? (2) interventions, i.e., will *X* happen if we make sure that event *Y* occurs? (3) counterfactuals, i.e., would *X* have occurred had event *Y* occurred, given context *Z*? Bochman and Lifschitz (2015) have shown that in the case of prediction questions, these can be answered using deductive inferences from a logical description of the causal world. Answers to intervention actions and counterfactuals rely on a submodel *M_*x*_* of a larger model *M*, where *x* is a variable of a set of variables *X* from *V*. This is a causal model obtained from *M* whereby the set of functions *F* are instead replaced by the function for variable *x* by the following set:


(3) $$F_{x}=\left\{f_{i}|V_{i}\notin X\right\}\cup \left\{X=x\right\}.$$

*F_*x*_* is therefore formed by deleting from *F* the functions *f_*i*_* that correspond to the members of set *X* and instead using the set of constant functions *X = x*. The submodel *M_*x*_* can be understood as the result of performing an action *X = x* on *M* that produces the minimal change required, whereby *X = x* holds true under any background exogenous variables *x*. This is Pearl’s submodel for evaluating counterfactuals that consider alternative situations, such as “Had *X* been *x* would *Y = y* still hold as true?”

Just as with the earlier examples given with RFT, Boolean logic propositions (propositional formulas) can now be proposed, except this time within a causal structural equation model, called a Boolean structural equation, and specifically within an RFT framework. Here it is assumed that a set of propositions is partitioned into a set of background exogenous propositions and a finite set of explainable endogenous propositions. A Boolean structural equation, expressed as *A = F*, shows that *F* is a propositional formula in which the endogenous proposition A does not appear. It also implies that a set of Boolean structural equations *A = F* should exist within a Boolean causal model for each endogenous proposition. A causal world solution of a Boolean causal model *M* is any propositional interpretation that satisfies the equivalences A↔F for all equations *A = F*, in the Boolean causal model *M*.

As an illustration of how RFT can be applied this way, consider the classic firing squad example (Pearl, 2000). Here, let *U, C, A, B* and *D*, respectively, represent the following five propositions: (1) “Court orders the execution,” (2) “Captain gives the signal,” (3) “Rifleman A shoots,” (4) Rifleman *B* shoots,” (5) “Prisoner dies.” This story of propositions can be formalized within the causal model *M*, where *U* is the only exogenous proposition within this model and this is stated as {C=U,A=C,B=C,D=A∨B}. There are only two solutions to this: the first being that all propositions are true, and the other is that all propositions are false. Static queries can be answered about this causal model, such as casual model *M* implies that ¬A→¬D, and this implication is satisfied by all the propositions of *M* when considered true about the causal world.

In a different scenario, a subset of the endogenous propositions *X* of the Boolean causal model *M* can be evaluated, whereby a truth-valued function *I* on *X* can be denoted by the submodel $M_{X}^{I}$ of *M*. Here, every equation *A = F* is replaced with *A = I(A)* where A∈X to form the submodel from *M*. To evaluate this new scenario, whereby in this scenario the captain did not give the signal, but the rifleman *A* shoots anyway, the prisoner dies, and rifleman *B* does not shoot, is given by the submodel $M_{\left\{A\right\}}^{I}$ of *M* with *I(A) = t*. This is the function of *A* being true, which means the proposition of the rifleman *A* shooting is also true. This can be denoted as {C=U,A=t,B=C,D=A∨B}. As this submodel implies ¬C→D (as rifleman *A* does shoot his gun in the case of the captain not giving the order to shoot) and ¬C→¬B (as riflemen *B* does not shoot his gun in the case of the captain not giving the order to shoot), and as these propositions are both true, this new situation is also justified.

This can be expanded more directly to the logical and set theory RFT interpretation of relational framing properties that have emerged from a logical theory of causal reasoning (relating to action and change) called causal calculus and emerged in the artificial intelligence (AI) literature but can also be applied to SEM. Largely based on work by Geffner (1994), causal calculus was introduced by McCain and Turner (1997) and generalized using first-order logic by Lifschitz (1997). The logical basis for causal calculus was first described by Bochman (2003), and its use as a general-purpose nonmonotonic formalism has also been explored (Bochman, 2004, 2007).

This allows for the establishment of propositional casual rules, such as *A* causes *B*, expressed as A⇒B, where both *A* and *B* are propositional formulas. A set of propositional causal rules makes up a propositional causal theory. Nonmonotonic semantics of a causal theory can also be expressed, where for a causal theory Δ and a set of *u* proposition, Δ(*u*) denotes a set of propositions that are caused by *u* in Δ, such as


(4) Δ(u)={B|A⇒B∈Δ,forsomeA∈u}

In an RFT example, the frame of causation can be explicitly represented within a structural equation model in the following set of propositions, which represents a modified version of the firing squad causal chain example and gives a maladaptive behavioral scenario. The propositions are as follows: (*A*) “A spider specialist explains that poisonous spiders exist in the local woods”; (*B*) “You fear poisonous spiders and now fear walking through the woods”; (*C*) “You avoid walking through the local woods”; (*D*) “You learn avoidance keeps you safe”; (*E*) “You avoid all experiences which make you feel unsafe.” This can be expressed through a causal chain, denoted as:


(5) $$\begin{array}{l} \Delta_{1}\left(u\right)=\left\{B|A\Rightarrow B\in \Delta_{1}\right\}\Rightarrow \Delta_{2}\left(u\right)=\left\{C|B\Rightarrow C\in \Delta_{2}\right\}\Rightarrow \\ \Delta_{3}\left(u\right)=\left\{D|C\Rightarrow D\in \Delta_{3}\right\}\Rightarrow \Delta_{4}\left(u\right)=\left\{E|D\Rightarrow E\in \Delta_{4}\right\} \end{array}$$

Here, there are four causal theories Δ and five propositions, whereby each causal theory consists of a set of two propositions and leads to another causal theory within the chain of the same number of propositions. This gives an example of how runaway thoughts have causal effects within an RFT network (and how possible self-isolative behavior occurs). These types of causal chains can be developed into RFT structure graphs along with functions such as ToF.

## Expressing causal logic through propositional ideographic graphs of graph theory

These sets of propositional logic expressed as causal theories within an RFT framework, as well as the other forms of mathematical relational frame logic mentioned, can be visualized within a graph using graph theory. This is provided to expand on current PBT approaches within the EEMM, which use process-based networks as part of their analysis at the individual ideographic level (Hofmann et al., 2021). There are existing graph theory packages available in *R* such as SEMgraph that specialize in modeling SEM graphs (Palluzzi and Grassi, 2021), as well as specialist non-SEM graph modeling approaches that focus specifically on RFT properties such as mutual entailment bidirectionality (Smith and Hayes, 2022). However, to date, no approach specifically brings about a propositional causal logic SEM RFT approach that comprehensively brings about complex relational framing dynamics within the causal networks that could be applied to PBT research.

Within graph theory, there are three types of path diagrams, which include (1) directed acyclic graphs (DAG) with values for each path within the graph in the form of beta coefficients $B_{jk}$, giving an indication of which paths or edges $\left(k\to j\right)$ within the graph have the most influence on some dependent measure (DV) within the network. In this case, all potential covariances are assumed null $\left(\psi_{jk}=0\right)$; (2) covariance models whereby only covariance can have a value greater than zero while coefficients can only equal zero $\left(B_{jk}=0\right)$; (3) Bow-free acrylic path graphs (BAP) have both bidirectional covariance relations$\left(k↔j\right)$ as well as acrylic directed edges $\left(k\to j\right)$. Bidirectional covariance only occurs when the *k* th and the j-th variables do not share a directed edge, so if $B_{jk}\neq 0$ then $\psi_{jk}=0$.

An SEM path diagram consists largely of linear regression equations and can be represented by a graph *G = (V, E)*, whereby the variables can be expressed as a set of nodes *V* and connections can be expressed as the set of edges *E*. The set of edges *E* can include both bidirectional edges k↔j if $k\in sib\left(j\right)$ to account for covariation, as well as directed k→j if $k\in pa\left(j\right)$ to account for the direct path coefficients and can be determined by the following:


(6) $$Y_{j}=\underset{k\in pa\left(j\right)}{\sum }B_{jk}Y_{k}+U_{j}\;j\in V$$

(7) $$Cov\left(U_{j};U_{k}\right)=\{\begin{array}{c} \psi_{jk}\\ 0 \end{array}\;\begin{array}{c} if\;j=k\;or\;k\;\in \;sib_{\left(j\right)}\;\\ otherwise \end{array}$$

Here, $Y_{j}$ is an observed variable, and $U_{j}$ is an unobserved error term. The regression coefficients are expressed as $B_{jk}$, while covariance is expressed as $\psi_{jk}$ and indicates that the errors are dependent. This assumes that there is an unobserved latent confounder between *k* and *j*.

Equations 6 and 7 can be written in a matrix form *Y = BY + U* and Cov(U)=ψ. Given some random variables with a zero-mean vector (u(θ)=0), the joint probability of *p* variables *Y* within a covariance matrix is given by:


(8) $$\sum \left(\theta \right)={\left(I-B\right)}^{-1}\psi {\left(I-B\right)}^{-T}$$

However, given that an idiographic approach is chosen here, and not a nomothetic one, certain ecological momentary assessments (EMA)-type questionnaires could capture the RFT properties (for a full discussion, see Supplementary material 8).

There are many ways EMA data can be analyzed, and one particularly useful approach at this ideographic level of assessment is the Group Iterative Multiple Model Estimation (GIMME; Gates and Molenaar, 2012). The algorithm is useful for time series data with at least 60 observations per person and currently fewer than 25 variables (Lane et al., 2019). The GIMME algorithm, at its core, searches for common and unique dynamic processes among individuals. It uses Lagrangian multiplier diagnostics (Sörbom, 1975), and it considers paths that significantly exist for the majority of individuals. This can be expanded with the idiographic filter and the Model Implied Instrumental Variables with Two-Stage Least Squares Estimation (MIIV-2SLS). Latent Variable—Group Iterative Multiple Model Estimation (LV-GIMME) has been suggested as optimal for individual-level time series data in psychological studies (Gates et al., 2020). This contrasts with the full information estimators such as the maximum likelihood (ML) of typical normative level SEM, which estimates the measurement model coefficients as influenced by values at the latent variable model level. MIIV-2SLS instead allows for an estimation of latent values across time at the individual level for all relations of the dynamic factor MODEL (DFM). As such, LV-GIMME operates within an SEM in a dynamic factor analysis framework for analysis of multivariate time series data (Molenaar, 1985), and this is given as follows:


(9) $$n_{t}=\alpha_{n}+An_{t}+\phi \xi_{t-1}+\zeta_{t}$$

Whereby *t* is time, and *t*–1 is variable at a previous time interval. Φ is the *P* × *P* matrix where *P* is the number of variables in the $\xi_{t-1}$. The matrix contains the vector autoregressive (VAR) effects (coefficients) for the $\xi_{t-1}$ variables that predict the endogenous $\eta_{t}$ values. Therefore, Φ contains the VAR (1) coefficients of how prior time point values relate to the subsequent point in time. In the original GIMME algorithm, $\xi_{t-1}$ and $\eta_{t}$ are simply observed variables, but they are latent variables in this SEM approach of LV-GIMME. The *P* × *P* -dimensioned A matrix has contemporaneous relations among the $\eta_{t}$ endogenous variables. $\zeta_{t}$ is the *P* × 1 vector that contains the errors or disturbances and is assumed to have a mean of zero. GIMME, therefore, allows the identification of structures of directed relations (paths) among the variables in the time-series data. The relations are the Φ and the *P* × *P* -diementionsed A matrices. Crucially, once the SEM path structure is established, graph modeling can follow similarly as described for the traditional SEM.

**Figure 6:**
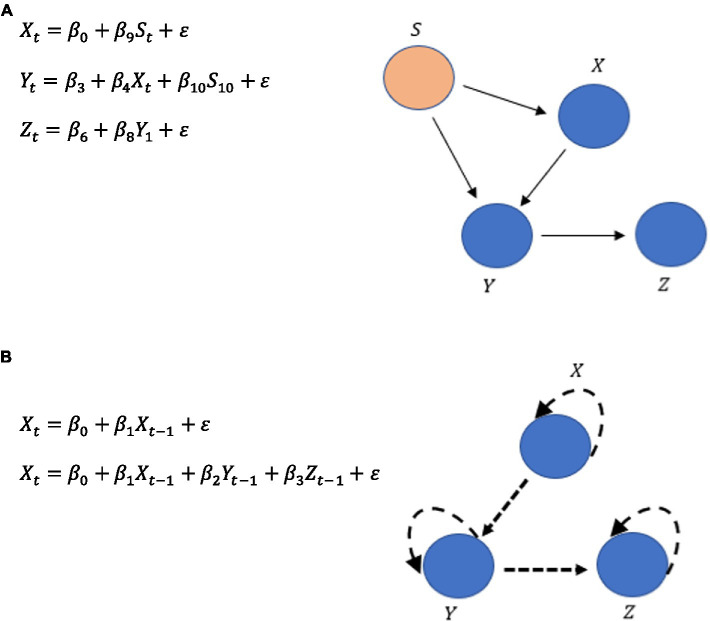
**(A)** Typical SEM pathways. **(B)** An illustration of the vector autoregressive updating in a time series ideographic SEM.

Notably, the difference between a traditional normative cross-sectional SEM and the idiographic SEM is that a traditional normative model would have specific directional paths such as those seen in Figure 6A, but an ideographic time series version such as GIMMIE would include a vector autoregression that updates the nodes given the additional data it has across time, as shown in Figure 6B.

**Figure 7:**
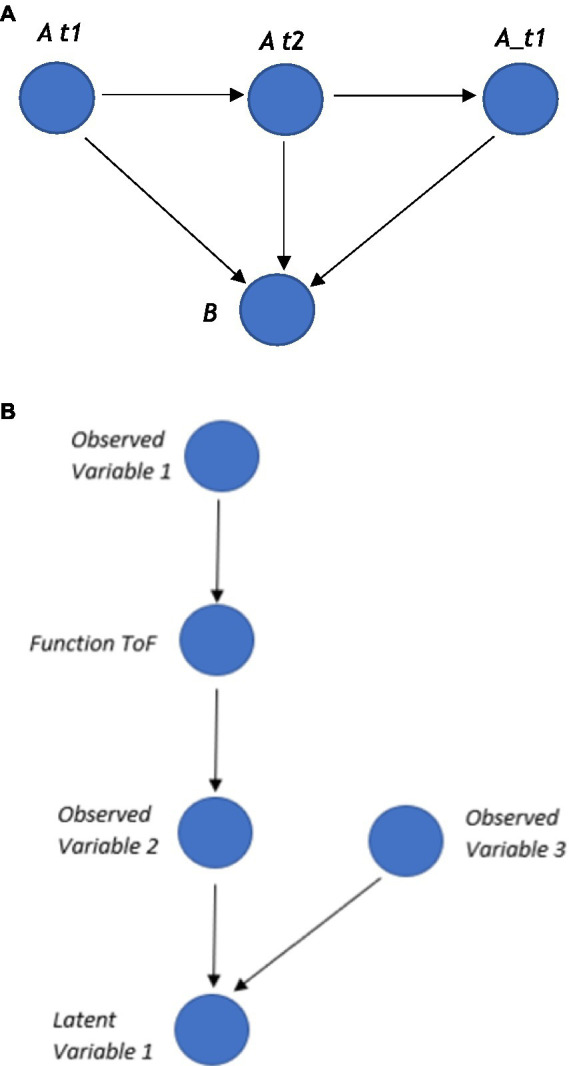
**(A)** Three time points *t*_1_, *t*_2_, *t*_3_ and two variables *A* and *B*. **(B)** A function node between two observed variables that express some relations between these observed variables, such as *latent variable=f(obsevered_variable_1,observed variable_2)*.

Crucially, these types of time series autoregressive SEMs can be represented within a graph, whereby nodes represent each variable, and the edges represent the relation between the nodes, such as the regression coefficients. In a time series, ideographic SEM additional information can be included about the different time points. One way to represent these time points within a graph is to create a separate node for each time point and connect an edge from each time node to a variable node (as shown in Figure 7A), which can be compressed later to the final autoregressive nodes for visual simplicity. Functions can also be added, such as a ToF, to these types of time-series SEMs, as shown in Figure 7B.

**Figure 8:**
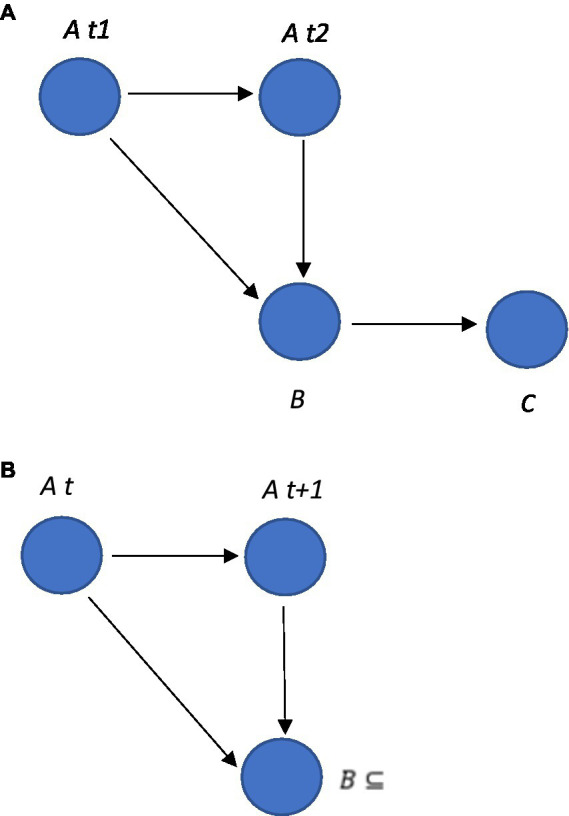
**(A)** An autoregressive VAR model within a graph. In this example, the graph includes three nodes, *A,B* and *C*, and four edges. The edges from node *A* to node *A* represent the autoregressive relationships, indicating that variable *A* depends on its own past values. The edge from node *B* to node *C* represents the relationship between these variables, indicating that *B* depends on *C*. **(B)** The edge from node *B* at time *t* + 1 to node *A* could be described as B(t+1)⊆A(t), which is interpreted as saying that the value of *B* at time *t* + 1 is a subset of the value of *A* at time *t*.

The autoregressive VAR model can be represented within the graph that illustrates the autoregressive nodal relationships, but in an SEM model as illustrated in Figure 8A. These types of autoregressive SEM graphs can be developed in Python, as indicated by the example code in Supplementary material 9.

It is also possible to apply logic and set theory in an SEM time-series autoregressive model such as GIMME. In an SEM, variables are often represented using sets, and the relationships between variables can be described using logical operators such as “AND” and “OR”. For example, it is possible to specify the relationship between two latent variables as a function (this in itself could have a function such as ToF or an operator such as AND expressed between two observed variables) using a logical expression such as:


$$\begin{array}{l} latent\_variable\left(t\right)=observed\_variable_{1}\left(t\right)\;\\ ANDobserved\_variable_{2}\left(t\right) \end{array}$$

This logical expression as a function could be interpreted in a way that the latent variable at the time (*t*) is only present when both of the observed variables are present at the time (*t*).

Set theory can be used in this type of autoregressive idiographic SEM to describe the relationship between variables (see Figure 8B). For example, it is possible to specify that a latent variable at time *t* + 1 is a subset of an observed variable at time *t* using set notation such as latentvariable(t+1)⊆observedvariable(t). This is interpreted as saying that the latent variable at time *t* + 1 includes all of the elements of the observed variables at time *t*, as well as possibly some additional elements. This naturally allows the RFT relational frames (such as ToF, opposition, and mutual entailment) to be expressed as logic and sets and modeled within this time series, autoregressive idiographic model approach.

## Overcoming logical paradoxes of self-reference and why the observer self needs to be specified mathematically outside of formal axioms

Although in the vast majority of cases specifying “self” leads to no problems in formal logic, perhaps one important observation is that self-reference within logic can, in some limited instances, lead to paradoxical statements that can be shown to be true but paradoxical in nature. This has important consequences when modeling the “self” within graphs. As an example of this, Hofstadter, in his books *I am Strange Loop* and *Gödel, Escher, Bach: An Eternal Golden Braid* (Hofstadter, 1979, 2007), referred to the properties of self-referential systems as demonstrated in Gödel’s incompleteness theorems (Gödel, 1931) and other areas as leading to paradoxes, which he refers to as strange loops. Penrose and Lucas made similar arguments, suggesting the mind and consciousness were beyond computation given Gödel’s incompleteness theorem (Lucas, 1961; Penrose and Mermin, 1990; Penrose, 1994). The full arguments for these are made in Supplementary material 10.

## The observer self, evolutionary Interface theory, and Markov kernels

One potentially useful avenue for modeling the observer self, and avoiding self-referential paradoxes is Hoffman and colleagues’ (Hoffman et al., 2015; Hoffman, 2016) interface theory of perception (ITP), whereby perceptual systems provide an organism-specific user interface promoted by evolutionary fitness and not veridical representations of the environment. This takes the ITP one step further by formally defining mathematically what they call a conscious agent (CA; Hoffman and Prakash, 2014; Fields et al., 2018), and this is applied as a minimally universally applicable formal model of conscious perception and behavior, potentially including the self as an observer to experience. CA is assumed to provide a universal representation of perceptual and cognitive processes in the context of ITP. There is also an assumption of consistency between CA and ITP, in the sense that a CA cannot respond (behave in response) to stimuli in the environment if the ITP does not detect them (Hoffman and Prakash, 2014). This mathematical expression of the CA that uses a Markov kernel may offer a useful fit for defining the conscious observer self in ACT and RFT, in order to prevent issues with self-reference paradoxes in strange loop logical systems. Notably, this can be implemented in graphs of graph theory alongside other logical RFT relational frame statements within a mathematically consistent and complete way, by utilizing a Markovian blanket, which would separate the observer self from the entanglement with logical propositional thoughts (i.e., self as content). Notably, conscious phenomenology in this approach is intended to model human phenomenology, whereby language plays a role in shaping phenomenology through concepts and categories that are verbally learned (from a Hebbian network) from the environment within the interface.

Crucially, ITP and CA can be regarded as ontologically neutral, given that perception and phenomenological experience are more generally bound to a fitness function rather than some form of mentalism. This aligns ITP away from a cognitive naïve realism position and more in line with the behavioral pragmatism of RFT that also holds an a-ontological position (Barnes-Holmes, 2005; Codd, 2015; Monestes and Villatte, 2015). This means that RFT could be interpreted through this (functional) interfacing approach based on evolutionary fitness rather than a cognitively vertical phenomenological experience. It is therefore conceptually a good fit for mathematically describing the observer self of ACT and RFT as well as the self within EEMM more generally.

The CA framework can be formally defined mathematically, which allows perceptions, decisions, and actions to be defined within a measurable space (definition 1 is of the Markov kernel, and definition 1 is of the CA):

Definition 1. Let 〈*B, B*〉 and 〈*C, C*〉 be measurable spaces, whereby *B* and *C* refer to σ-algebra of measurable space called events and represent a collection of subsets *B* and *C*, respectively. Then equip the unit interval *0, 1* with its Borel σ-algebra. The function K:B×C→0,1 is a Markovian kernel from *B* to *C* if

(i) For each measurable set E∈B, the function K(⋅,E):B→[0,1] enacted by b↦K(b,E) is a measurable function.
(ii) For each b∈B, the function K(b,⋅) enacted by F↦K(b,F),F∈C is a probability measure on *C*.

**Figure 9:**
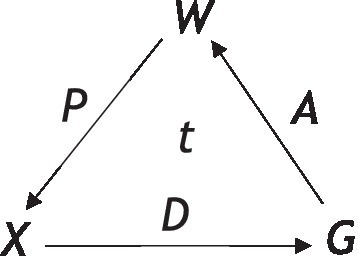
Illustration within a three-node graph, the conscious agent (CA). *W* (world), *X* (experience), and *G* (conscious action) are measurable sets, while *P* (perceive), *D* (decision), and *A* (action) are Markovian kernels, and t is an integer parameter [Reprinted with permission from Elsevier (Fields et al., 2018)].

A CA can be represented as a directed graph, as illustrated in Figure 9. The graph demonstrates a cyclic process, whereby a kernel D:X×G→G can be thought of in the following way: for each instantiation $g_{0}$ of *G* in the immediately previous cycle, and the current instantiation of $x\in \;X,D\;\left(x,g_{0};\cdot \right)$ gives the probability distribution of the g∈G instantiated in the next step. Kernels *A* and *P* are instantiated in the same way. To put it more formally

Definition 2. Let 〈*W, W*〉, 〈*X, X*〉 and 〈*G, G*〉 be measurable spaces. Let *P* be a Markovian kernel P:W×X→X, *D* be a kernel D:X×G→G, and *A* be a kernel A:G×W→W. A CA is a 7 tuple [(X,X),(G,G)(W,W),P,D,A,t], where *t* is a positive integer parameter.

*W* is interpreted as elements of the world, *X* and *G* are interpreted as representing (consisting of tokens) different conscious experiences and actions, respectively. Kernels *P*, *D*, and *A* represent perception, decision, and action (behavior) operators. Any operator that changes the state of *X* (conscious experience) is regarded as “perception,” any operator that changes the state of *G* (conscious action) is regarded as a “decision,” and any operator that changes the state of *W* (world state) is regarded as an “action.” The perception set *X* takes all phenomenological representations of experience, not just visual, i.e., all modalities. Similarly, as set *G* and kernel *A* are also multimodal, perception can be viewed as an action performed by the world. When states *W*, *X*, and *G* change, kernels *P*, *D*, and *A* act in response, respectively. Decisions *D* and actions *A* of the CA are assumed to be freely chosen and not deterministic (particularly when directed by the observer self rather than self as content), and as such, these operators are treated as stochastic in the general case. CA-specific proper time is denoted by *t* and is incrementally “ticking” concurrently with the action of decisions *D*, and change of state of *X* (hence applicable for ideographic time series analysis such as in PBT). There are no assumptions about what *X* contains, such as containing tokens representing the value of *t* or the elements of *G*. Further details of this approach are given in Supplementary material 11.

## Embodied cognition, entropy, Markov blanket, CA, and self

An important construct of the EEMM is the level of psychobiology and how this relates to, for instance, the dimension of “self.” A conscious agent of “self” would receive many inputs from brain neurons and interoceptively through the body. Embodied cognition and interception play a major role in shaping a meta-representation of “self.” These are relevant to ACT and RFT concepts as, for example, ACT embodied knowledge (such as being aware, engaged, and open) has been identified as relevant in participants naïve to ACT and used to describe (or score) several bodily postures (Falletta-Cowden et al., 2022) that could be interoceptively related. Embodiment, such as interoceptive awareness and related vagal function, has been shown to play an important role in emotional regulation and coping (Pinna and Edwards, 2020). Full details of interoception and how this relates to brain structures that form a representation of “self” are given in Supplementary material 12. Notably, the underlying learning is considered Hebbian in nature, as described in a previous study (Edwards et al., 2022).

**Figure 10:**
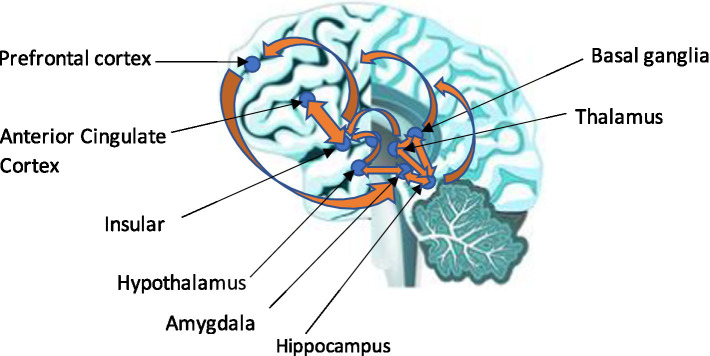
A combined lateral and medial view of the limbic, insular cortex, and prefrontal cortex axes. The biopsychological level, neuro-integrated, and embodied network.

**Figure 11:**
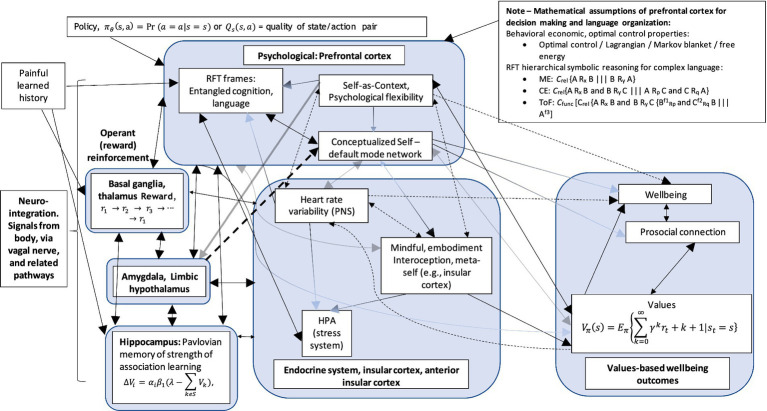
A potential example of a biopsychological level, neuro-integrated, and embodied network (NIEN). (1) Large arrowheads = greater excitatory effect; (2) Fading arrowhead = inhibitory effect; (3) Dashed line with directional arrowhead = mediating association; (4) Dashed line with bidirectional arrow heads = mediating or moderation association.

Similar connectivity has been found with major depressive disorder (MDD). For example, MDD, which is also a disorder of the regulation of mood and emotion, has been assumed to be the result of cortical–limbic circuitry (Kennedy et al., 2001). A review of the evidence suggested that abnormalities in the structure and function of the prefrontal cortex, anterior cingulate, hippocampus, and amygdala were responsible for depression (Davidson et al., 2002). It is perhaps interesting that the hippocampus was mentioned, as this is the area where associational learning takes place, such as Pavlovian conditioning, and may relay information to the prefrontal cortex such as situation “A” is scary. The prefrontal cortex can, perhaps, then make decisions about what situations “A” should be avoided. Expanding on this, the hippocampus is thought to provide context-dependent information, as fear extinction is context-dependent, and is thought to involve the inhibitory control of the prefrontal cortex over amygdala-based fear processes, whereby hippocampal-based contextual information is integrated with the prefrontal–amygdala circuitry (Sotres-Bayon et al., 2004). Central brain components and relevant feedback loops are illustrated in Figure 10. Figure 11 illustrates these connections leading to goals and values. Full mathematical accounts for generic relational frames, values, and associations can be found in the study by Edwards (2021).

There seem to be very clear neurological pathways between the emergence of the embodied self from interoceptive signals (Seth, 2013), the default mode network, which is thought to process self-referential thought, and depression (Sheline et al., 2009; Paulus and Stein, 2010; Lemogne et al., 2012; Zhu et al., 2012; Koban et al., 2021). The default mode network is particularly important as the processing of self-referential thought includes self-reflection (perspective-taking), future things, ruminating and remembering events in the past, which are all deictic RFT processes, with very relevant neurological pathways to self as content and the observer self. Interestingly, drug compounds that low the processing of the default mode network such as psilocybin lead to participants reporting the phenomenological experience of their sense of self such as ego (the self-stories, narrative self as content) dissolving, and this includes the boundary between their self and the environment (Carhart-Harris et al., 2013; Lebedev et al., 2015; Pollan, 2015; Barrett and Griffiths, 2018), and mindfulness exercises have also been found to dissolve self to some degree (Cooper et al., 2022), such as the body scan have led to similar effects such as dissolving bodily boundaries of self (Hanley et al., 2020) and rigid patterns of defensive-self (Garland et al., 2017). For full details of how interoception, embodied cognition of self, relates to entropy and predictive coding and some of these ideas that emerged decades ago, when the physicist Schrödinger (1942) in his seminal book, asked “*What Is Life?”* (see Supplementary material 13).

**Figure 12:**
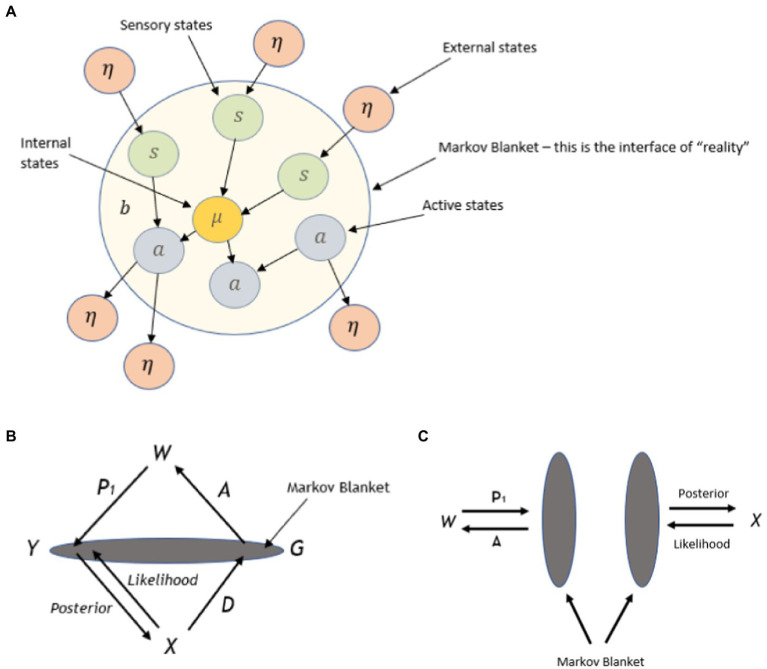
**(A)** A Markov blanket *b* that highlights the exchange of information with its surroundings whereby external states (variables) *η* are conditionally independent of internal states *μ*. Note: Markov blanket = *b*; *η* = external states; *μ* = internal states; *s* = sensory states; *a* = active states. **(B)** *P* in a CA framework with a Markov blanket, with *P*_2_ in the CEP formalism, produces a four-node graph, replacing the three-node graph of the typical CA (Figure 9). Bidirectional indirect interaction between *W* and *X* *via* their proximal surfaces is demonstrated in B and C [Reprinted with permission from Elsevier (Fields et al., 2018)].

However, to explore how biological systems act through Bayesian inference as suggested by predictive coding to reduce entropy specifically (Kuchling et al., 2020), Lagrangian mechanics need to be explained. This is a problem of dynamical systems and identifying a Lyapunov function can be used to analyze and solve any dynamic system using the fundamental theorem of vector calculus—the Helmholtz decomposition. This can be used to characterize the general flow of systemic states toward the convergence of a nonequilibrium steady state. Markov blankets are then introduced, which separate the internal and external states of a system, whereby the Markov blanket is comprised of sensory and active states (see Figure 12A). Notably, the Markov blanket also acts as the evolutionary interface based on fitness that separates and disassociates the observer self from the self as the content of the world. Using the partition of the Markov blanket, the Lyapunov function can be replaced with variational free energy to solve the equations of internal and external states and therefore characterize self-organization as equilibrium states that can be portioned into a system (i.e., the internal state of a Markov blanket) and its external environment.

In terms of updating the system, the active inference is utilized. This is based on Bayesian inference, which uses Bayes’ theorem as a statistical approach whereby the probability of a hypothesis is updated with respect to some measured evidence gained from the sensorium or the environment. Bayes’ theorem can be given as $\left(A|B\right)=\frac{P\left(B|A\right)P\left(A\right)}{P\left(B\right)}$, where the conditional probability of an unobservable event *A* given some observable event *B* P(A|B) (the inferred probability of an event *A*) is called the *posterior* belief. Conversely, P(B|A) reflects the probability of event *B* given the occurrence of event *A*, *P(A)* is the *prior* belief, while *P(B)* is the marginal likelihood of evidence. In Bayesian inference, the Bayes theorem is used to accumulate information about an unobservable (or hidden) state by sampling measurable states, which is known as Bayesian belief updating as it converts prior beliefs into posterior beliefs based on its generative model, P(B|A)=P(B|A)P(A). Thus, the updating dynamics of a system can be described as the probability of likelihood assigned to sensory observations and prior (predictive expectation) beliefs combined. Hence, a self-organizing system can be thought of as an information processing system that infers unobservable hidden states of its environment by comparing sensory samples with predictions of sensory input and updating its expectations about the causes of that input.

For a complete understanding of the Bayesian interpretation of nonequilibrium, steady state dynamics, a brief mathematical foundation, and an overview of the Helmholtz decomposition and Lyapunov function are given. Dynamics can be formulated by functions that play the role of a Lyapunov function, such as those illustrated in classical mechanics with dissipative aspects. The same results can also be derived through the Fokker-Plank equation in generalized coordinates of motion. This shows that the Lyapunov function is simply the negative log probability of a state being occupied at a nonequilibrium steady state. In this way, the flow of states at a nonequilibrium steady state can be placed on the same gradient as the quantity that is minimized (entropy) by the Bayesian belief updating (see Supplementary material 14 for details).

This approach exploits the principle of least action integral of Lagrangian mechanics and turns it into an integration over the self-information of states, known as entropy in information theory. Crucially, the principle of least action manifests itself into the principle of least internal entropy for systems that possess a (dissipative) random dynamical attractor and, therefore, obtain a nonequilibrium steady state. The specific structure of the system or model *m* that underwrites the Bayesian inference is called the Markov blanket. The CA for self, utilizing a Markov blanket, therefore may be one way to structure the observer self from the world state which produced a four-node graph (see Figure 12B), and this forms a bidirectional indirect interaction between *W* and *X* *via* their proximal surfaces (see Figure 12C).

The Markov blanket consists of active and sensory states, whereby (1) internal states govern the active states but affect the external states and (2) external states govern sensory states but affect internal states (Kirchhoff et al., 2018). The free energy (or prediction error) can be minimized in two ways: either by (1) perception, which is the updating of the prediction based on the sensation, or (2) action, which is the changing of the sensation so that it matches the prediction (Seth, 2013; Holmes and Nolte, 2019). The Markov blanket *b* consists of sensory states *s* that affect but are not affected by internal states μ as well as active states *a* that affect but are not affected by external states η.

**Figure 13:**
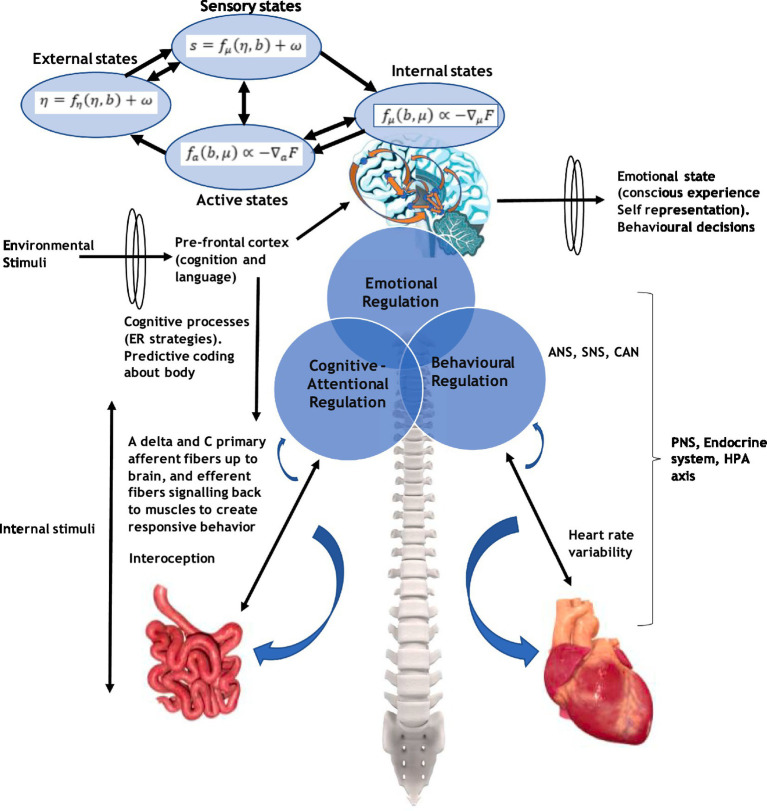
A schematic representation of the ensemble dynamics and Markov blankets, and the bottom-up and top-down relationships between interoception, parasympathetic vagal tone, and central self-regulatory centers.

As such, *b* is defined as the set of variables (states) that renders μ conditionally independent from η. This can be donated mathematically as described in Supplementary material 15. In addition to this, the sensory states *s* children in the form of active states *a* mediate the influence of internal states μ on external states η (see Figure 3 of this directional relation). These ensemble dynamics and Markov blankets can be seen in Figure 13 and depicted more generally with the fully embodied interoceptive system, which depicts the bottom-up and top-down relationships between interoception, parasympathetic vagal tone, and central self-regulatory centers.

**Figure 14:**
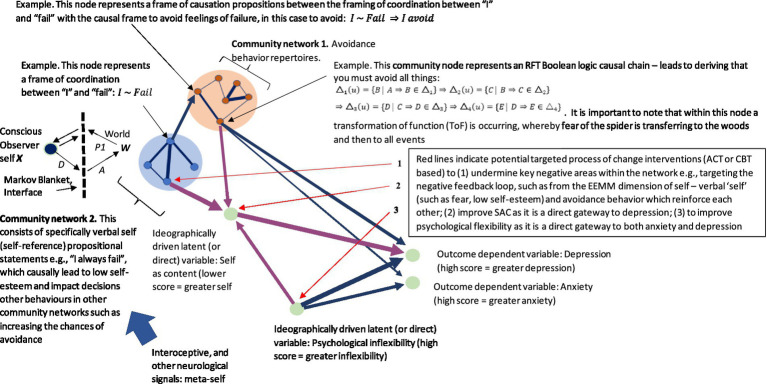
Illustration of RFT-derived Boolean logic causal chains applied to an SEM within a graph of nodes and edges *G = (V, E)*. Each node within a community represents a propositional causal chain (causality) or other forms of relational framing. Wider-directed lines indicate larger coefficients. Dark blue lines indicate a positive coefficient, whereas purple lines indicate negative coefficients. Note: It is plausible to assume these dynamics occur in the frontal cortex (whereby signals are fed from lower-level brain structures and body) as part of hierarchical pattern RFT relational framing (N-framing).

This finalizes the overall N-frame system, whereby the Markov blanket separating the observer self and representing the evolutionary fitness interface with the world can be depicted within the relational frame ideographic graphs (see Figure 14). This can then be utilized for PBT analysis and overcoming some of the strange self-referential loops (as the observer self is outside of the system that describes it). The Markov blanket, which acts as the interface to the world, represents the set of variables that are related to, but not exclusively, self-reference (i.e., self as content), shielding from the effects of other variables at the observer-self that may cause paradoxes. Similarly, the CA is the self-observer outside of the Markov blanket, and therefore a self-referential system that is described mathematically as a Markov kernel can describe the probability of transitions from state to state in a Markov process. By representing self-reference as a Markov process, it may be possible to use the Markov kernel to model the transition between states of self-reference and non-self-reference, thus possibly avoiding paradoxes that arise from self-reference, which includes some function *f* of reinforcement feedback from the world *w* as specified in the previous study as a Markov model (Edwards, 2021).

In conclusion, though this framework has not been developed specifically for clinical purposes, it gives a possible way to bring about an idiographic functional contextual RFT approach to clinical assessment and treatment specification in the form of process-based therapy. It specifically does this by giving an ontologically neutral and functionally context-dependent evolutionary interface solution to extracting relational frames and scaling with PBT graphs. It does this by giving a mathematical description to RFT through propositional logic and then ideographically scaling this into complex network graphs, whereby clinicians could utilize these graphs to explore areas within the network whereby negative relational frame loops occur (such as a fear transformation of function) that leads to cognitive fusion, low self-esteem, avoidance, etc. Once these negative relational frame loop areas are identified, the therapist can then target these areas with interventions that are likely to undermine the dominance they hold over the network, such as value identification, mindfulness, and cognitive defusion tasks. An example of such targeting is given in Figure 14, whereby the red lines indicate the potential targeted process of change interventions (ACT or CBT based) to (1) undermine key negative areas within the network, e.g., targeting the negative feedback loop, such as from the EEMM dimension of self—verbal “self” (such as fear, low self-esteem) and avoidance behavior which reinforces each other; (2) improve SAC as it is a direct gateway to depression; (3) to improve psychological flexibility as it is a direct gateway to both anxiety and depression. Using this approach should require a therapist with minimal training (video or workshop) to use some ecological momentary assessment of relational frames (instances of thought entanglement, etc.) application that processes that data as described, that would assess variables at several points in the day over a week (or more), and that automatically visualizes the data within network graphs. This interface solution for self may also be an important contribution to the AI literature, whereby some form of self-reference, self-reflection, or self-awareness (similar to a default mode network) may be possible as they evolve to become more human-like and a natural extension to a previous study in this area (Edwards et al., 2022).

## Data availability statement

Publicly available datasets were analyzed in this study. The datasets are available at the following link: https://github.com/DarrenEdwards111/Example-relational-network-code.

## Author Contributions

The author DE confirms being the sole contributor of this work and has approved it for publication.

## Conflict of interest

The author declares that the research was conducted in the absence of any commercial or financial relationships that could be construed as a potential conflict of interest.

## Publisher’s note

All claims expressed in this article are solely those of the authors and do not necessarily represent those of their affiliated organizations, or those of the publisher, the editors and the reviewers. Any product that may be evaluated in this article, or claim that may be made by its manufacturer, is not guaranteed or endorsed by the publisher.

## Footnotes

^1^All Python code is available on GitHub: https://github.com/DarrenEdwards111/Example-relational-network-code

## References

[ref1] American Psychiatric Association. (2013). Diagnostic and statistical manual of mental disorders: DSM-5: 10 Washington, DC: American psychiatric association.

[ref2] Atkins P. W. Wilson D. S. Hayes S. C. (2019). Prosocial: Using evolutionary science to build productive, equitable, and collaborative groups Vol. 33, New Harbinger Publications.

[ref3] Barnes-Holmes D. (2005). Behavioral pragmatism is a-ontological, not antirealist: a reply to Tonneau. Behav. Philos., 33, 67–79.

[ref4] Barnes-Holmes D. Hayes S. C. Roche B. (2001). Relational frame theory: A post-Skinnerian account of human language and cognition. New York, NY: Plenum Publishers. 10.1016/s0065-2407(02)80063-5 11605362

[ref5] Barrett F. S. Griffiths R. R. (2018). Classic hallucinogens and mystical experiences: phenomenology and neural correlates. Curr. Top. Behav. Neurosci. 36, 393–430. doi: 10.1007/7854_2017_474 28401522 PMC6707356

[ref6] Bennett A. T. Cuthill I. C. (1994). Ultraviolet vision in birds: what is its function? Vis. Res. 34, 1471–1478. doi: 10.1016/0042-6989(94)90149-X 8023459

[ref7] Blackledge J. T. (2003). An introduction to relational frame theory: basics and applications. Behav. Anal. Today 3, 421–433. doi: 10.1037/h0099997

[ref8] Bochman A. (2003). A logic for causal reasoning. Paper presented at the IJCAI.

[ref9] Bochman A. (2004). A causal approach to nonmonotonic reasoning. Artif. Intell. 160, 105–143. doi: 10.1016/j.artint.2004.07.002

[ref10] Bochman A. (2007). A causal theory of abduction. J. Log. Comput. 17, 851–869. doi: 10.1093/logcom/exm045

[ref11] Bochman A. Lifschitz V. (2015). Pearl's causality in a logical setting. Paper presented at the Proceedings of the AAAI Conference on Artificial Intelligence, 29

[ref12] Carhart-Harris R. L. Leech R. Erritzoe D. Williams T. M. Stone J. M. Evans J.. (2013). Functional connectivity measures after psilocybin inform a novel hypothesis of early psychosis. Schizophr. Bull. 39, 1343–1351. doi: 10.1093/schbul/sbs117, PMID: 23044373 PMC3796071

[ref13] Ciarrochi J. Sahdra B. Hofmann S. G. Hayes S. C. (2022). Developing an item pool to assess processes of change in psychological interventions: the process-based assessment tool (PBAT). J. Contextual Behav. Sci. 23, 200–213. doi: 10.1016/j.jcbs.2022.02.001

[ref14] Codd R. T. III (2015). The functional contextual a-ontological stance and bas C. van Fraassen’s constructive empiricism. Journal of contextual. Behav. Sci. 4, 215–219. doi: 10.1016/j.jcbs.2015.05.004

[ref15] Cooper A. C. Ventura B. Northoff G. (2022). Beyond the veil of duality—topographic reorganization model of meditation. Neurosci. Conscious. 2022:niac013. doi: 10.1093/nc/niac013 36237370 PMC9552929

[ref16] Darwin C. (1859). The origin of species by means of natural selection. 6th Edn Project Gutenburg.

[ref17] Davidson R. J. Pizzagalli D. Nitschke J. B. Putnam K. (2002). Depression: perspectives from affective neuroscience. Annu. Rev. Psychol. 53, 545–574. doi: 10.1146/annurev.psych.53.100901.135148 11752496

[ref01] Dickinson S. J. Pizlo - Singh Z. M. Hoffman D. D. (2013). “Natural selection and shape perception” in Shape perception in Human and Computer Vision, SJ Dickinson, Z Pizlo (Springer), 171–185.

[ref18] Duchamp-Viret P. Chaput M. Duchamp A. (1999). Odor response properties of rat olfactory receptor neurons. Science 284, 2171–2174. doi: 10.1126/science.284.5423.2171 10381881

[ref19] Edwards D. J. (2021). Ensuring effective public health communication: insights and modeling efforts from theories of behavioral economics, heuristics, and behavioral analysis for decision making under risk. Front. Psychol. 12:715159. doi: 10.3389/fpsyg.2021.715159, PMID: 34721162 PMC8548420

[ref20] Edwards D. J. McEntaggart C. Barnes-Holmes Y. (2022). A functional contextual account of background knowledge in categorization. Front. Psychol. 13:745306. doi: 10.3389/fpsyg.2022.745306 35310283 PMC8924495

[ref21] Falletta-Cowden N. Smith P. Hayes S. C. Georgescu S. Kolahdouzan S. A. (2022). What the body reveals about lay knowledge of psychological flexibility. J. Clin. Med. 11:2848. doi: 10.3390/jcm11102848, PMID: 35628974 PMC9144916

[ref22] Fields C. Hoffman D. D. Prakash C. Singh M. (2018). Conscious agent networks: formal analysis and application to cognition. Cogn. Syst. Res. 47, 186–213. doi: 10.1016/j.cogsys.2017.10.003

[ref23] Friston K. (2010). The free-energy principle: a unified brain theory? Nat. Rev. Neurosci. 11, 127–138. doi: 10.1038/nrn2787 20068583

[ref24] Garland E. L. Hanley A. W. Baker A. K. Howard M. O. (2017). Biobehavioral mechanisms of mindfulness as a treatment for chronic stress: an RDoC perspective. Chronic Stress 1:247054701771191. doi: 10.1177/2470547017711912 PMC5565157 28840198

[ref25] Gates K. M. Fisher Z. F. Bollen K. A. (2020). Latent variable GIMME using model implied instrumental variables (MIIVs). Psychol. Methods 25, 227–242. doi: 10.1037/met0000229, PMID: 31246041 PMC6933098

[ref26] Gates K. M. Molenaar P. C. (2012). Group search algorithm recovers effective connectivity maps for individuals in homogeneous and heterogeneous samples. NeuroImage 63, 310–319. doi: 10.1016/j.neuroimage.2012.06.026, PMID: 22732562

[ref27] Geffner H. (1994). Causal default reasoning: Principles and algorithms. Paper presented at the AAAI.

[ref28] Gehring W. J. (2014). The evolution of vision. Wiley Interdiscip. Rev. Dev. Biol. 3, 1–40. doi: 10.1002/wdev.96 24902832

[ref29] Geisler W. S. Diehl R. L. (2003). A Bayesian approach to the evolution of perceptual and cognitive systems. Cogn. Sci. 27, 379–402. doi: 10.1207/s15516709cog2703_3

[ref30] Gödel K. (1931). Über formal unentscheidbare Sätze der Principia Mathematica und verwandter Systeme I. Monatsh. Math. 38-38, 173–198. doi: 10.1007/BF01700692

[ref31] Gross A. C. Fox E. J. (2009). Relational frame theory: an overview of the controversy. Anal. Verbal Behav. 25, 87–98. doi: 10.1007/BF03393073, PMID: 22477432 PMC2779078

[ref32] Hanley A. W. Dambrun M. Garland E. L. (2020). Effects of mindfulness meditation on self-transcendent states: perceived body boundaries and spatial frames of reference. Mindfulness 11, 1194–1203. doi: 10.1007/s12671-020-01330-9, PMID: 33747250 PMC7968136

[ref33] Hayes S. C. Ciarrochi J. Hofmann S. G. Chin F. Sahdra B. (2022). Evolving an idionomic approach to processes of change: towards a unified personalized science of human improvement. Behav. Res. Ther. 156:104155. doi: 10.1016/j.brat.2022.104155 35863243

[ref34] Hayes S. C. Hofmann S. G. (2018). Process-based CBT: The science and core clinical competencies of cognitive behavioral therapy, New Harbinger Publications.

[ref35] Hayes S. C. Hofmann S. G. Ciarrochi J. (2020). A process-based approach to psychological diagnosis and treatment: the conceptual and treatment utility of an extended evolutionary meta model. Clin. Psychol. Rev. 82:101908. doi: 10.1016/j.cpr.2020.101908 32932093 PMC7680437

[ref36] Hayes S. C. Hofmann S. G. Stanton C. E. Carpenter J. K. Sanford B. T. Curtiss J. E.. (2019). The role of the individual in the coming era of process-based therapy. Behav. Res. Ther. 117, 40–53. doi: 10.1016/j.brat.2018.10.005, PMID: 30348451

[ref37] Hayes S. C. Strosahl K. D. Bunting K. Twohig M. Wilson K. G. (2004). “What is acceptance and commitment therapy?” in A practical guide to acceptance and commitment therapy eds. S. C. Hayes, K. D. Strosahl (Springer), 3–29.

[ref38] Hayes S. C. Strosahl K. D. Wilson K. G. (1999). Acceptance and commitment therapy: Guilford press: New York.

[ref39] Hayes S. C. Strosahl K. D. Wilson K. G. (2011). Acceptance and commitment therapy: The process and practice of mindful change, Guilford press.

[ref40] Hoffman D. D. (2016). The interface theory of perception. Curr. Dir. Psychol. Sci. 25, 157–161. doi: 10.1177/0963721416639702

[ref41] Hoffman D. D. (2019). The case against reality: How evolution hid the truth from our eyes. London: Allen Lane.

[ref42] Hoffman D. D. Prakash C. (2014). Objects of consciousness. Front. Psychol. 5:577. doi: 10.3389/fpsyg.2014.00577 24987382 PMC4060643

[ref43] Hoffman D. D. Singh M. (2012). Computational evolutionary perception. Perception 41, 1073–1091. doi: 10.1068/p7275 23409373

[ref44] Hoffman D. D. Singh M. Mark J. (2013). Does evolution favor true perceptions? Paper presented at the Human Vision and Electronic Imaging XVIII.

[ref45] Hoffman D. D. Singh M. Prakash C. (2015). The interface theory of perception. Psychon. Bull. Rev. 22, 1480–1506. doi: 10.3758/s13423-015-0890-8 26384988

[ref46] Hofmann S. G. Barber J. P. Salkovskis P. Wampold B. E. Rief W. Ewen A.-C. I.. (2022). What is the common ground for modern psychotherapy? A discussion paper based on EACLIPT’s 1st webinar. Clin. Psychol. Europe 4, 1–8. doi: 10.32872/cpe.8403 PMC9667344 36397744

[ref47] Hofmann S. G. Hayes S. C. Lorscheid D. N. (2021). Learning process-based therapy: A skills training manual for targeting the core processes of psychological change in clinical practice Oakland, California: New Harbinger Publications.

[ref48] Hofstadter D. R. (1979). Gödel, escher, bach. Basic books: New York.

[ref49] Hofstadter D. R. (2007). I am a strange loop. New York: Basic books.

[ref50] Holmes J. Nolte T. (2019). Surprise and the bayesian brain: implications for psychotherapy theory and practice. Front. Psychol. 10:592. doi: 10.3389/fpsyg.2019.00592, PMID: 30984063 PMC6447687

[ref51] Jones G. Teeling E. C. Rossiter S. J. (2013). From the ultrasonic to the infrared: molecular evolution and the sensory biology of bats. Front. Physiol. 4:117. doi: 10.3389/fphys.2013.00117 23755015 PMC3667242

[ref52] Keller A. Vosshall L. B. (2008). Better smelling through genetics: mammalian odor perception. Curr. Opin. Neurobiol. 18, 364–369. doi: 10.1016/j.conb.2008.09.020, PMID: 18938244 PMC2590501

[ref53] Kennedy S. H. Evans K. R. Krüger S. Mayberg H. S. Meyer J. H. McCann S.. (2001). Changes in regional brain glucose metabolism measured with positron emission tomography after paroxetine treatment of major depression. Am. J. Psychiatr. 158, 899–905. doi: 10.1176/appi.ajp.158.6.899, PMID: 11384897

[ref54] Kirchhoff P. Palacios F. Kiverstein. (2018). The Markov blankets of life: autonomy, active inference and the free energy principle. J. R. Soc. Interface 15:20170792. doi: 10.1098/rsif.2017.0792, PMID: 29343629 PMC5805980

[ref55] Koban L. Gianaros P. J. Kober H. Wager T. D. (2021). The self in context: brain systems linking mental and physical health. Nat. Rev. Neurosci. 22, 309–322. doi: 10.1038/s41583-021-00446-8, PMID: 33790441 PMC8447265

[ref56] Kuchling F. Friston K. Georgiev G. Levin M. (2020). Morphogenesis as Bayesian inference: a variational approach to pattern formation and control in complex biological systems. Phys Life Rev 33, 88–108. doi: 10.1016/j.plrev.2019.06.001, PMID: 31320316

[ref57] Lane S. T. Gates K. M. Pike H. K. Beltz A. M. Wright A. G. (2019). Uncovering general, shared, and unique temporal patterns in ambulatory assessment data. Psychol. Methods 24, 54–69. doi: 10.1037/met0000192, PMID: 30124300 PMC6433550

[ref58] Lebedev A. V. Lövdén M. Rosenthal G. Feilding A. Nutt D. J. Carhart-Harris R. L. (2015). Finding the self by losing the self: neural correlates of ego-dissolution under psilocybin. Hum. Brain Mapp. 36, 3137–3153. doi: 10.1002/hbm.22833, PMID: 26010878 PMC6869189

[ref59] Lemogne C. Delaveau P. Freton M. Guionnet S. Fossati P. (2012). Medial prefrontal cortex and the self in major depression. J. Affect. Disord. 136, e1–e11. doi: 10.1016/j.jad.2010.11.034 21185083

[ref60] Lifschitz V. (1997). On the logic of causal explanation. Artif. Intell. 96, 451–465. doi: 10.1016/S0004-3702(97)00057-X

[ref61] Lucas J. R. (1961). Minds, Machines and Gödel1. Philosophy 36, 112–127. doi: 10.1017/S0031819100057983

[ref62] Maloney L. T. Zhang H. (2010). Decision-theoretic models of visual perception and action. Vis. Res. 50, 2362–2374. doi: 10.1016/j.visres.2010.09.031, PMID: 20932856

[ref63] Mark J. T. Marion B. B. Hoffman D. D. (2010). Natural selection and veridical perceptions. J. Theor. Biol. 266, 504–515. doi: 10.1016/j.jtbi.2010.07.020, PMID: 20659478

[ref64] Marr D. (1982). Vision: Freeman, San Fransico.

[ref65] Marr D. (2010). Vision: A computational investigation into the human representation and processing of visual information Cambridge, Massachusetts: MIT press.

[ref66] McCain N. Turner H. (1997). Causal theories of action and change. Paper presented at the AAAI/IAAI.

[ref67] Molenaar P. (1985). A dynamic factor model for the analysis of multivariate time series. Psychometrika 50, 181–202. doi: 10.1007/BF02294246

[ref68] Monestes J.-L. Villatte M. (2015). Humans are the selection criterion in psychological science, not “reality”: a reply to Herbert and Padovani. J. Contextual Behav. Sci. 4, 210–211. doi: 10.1016/j.jcbs.2015.06.003

[ref69] Mulhern T. Stewart I. McElwee J. (2018). Facilitating relational framing of classification in young children. J. Contextual Behav. Sci. 8, 55–68. doi: 10.1016/j.jcbs.2018.04.001

[ref70] Nowak M. A. (2006). Evolutionary dynamics: Exploring the equations of life, Cambridge, Massachusetts: Harvard university press.

[ref71] Ollivier F. Samuelson D. Brooks D. Lewis P. Kallberg M. Komáromy A. (2004). Comparative morphology of the tapetum lucidum (among selected species). Vet. Ophthalmol. 7, 11–22. doi: 10.1111/j.1463-5224.2004.00318.x, PMID: 14738502

[ref72] Palluzzi F. Grassi M. (2021). SEMgraph: An R package for causal network analysis of high-throughput data with structural equation models arXivpreprint arXiv:2103.08332. 10.1093/bioinformatics/btac567 36040154

[ref73] Palmer S. E. (1999). Vision science: Photons to phenomenology, Cambridge, Massachusetts: MIT press.

[ref74] Paulus M. P. Stein M. B. (2010). Interoception in anxiety and depression. Brain Struct. Funct. 214, 451–463. doi: 10.1007/s00429-010-0258-9, PMID: 20490545 PMC2886901

[ref75] Pearl J. (1988). Probabilistic reasoning in intelligent systems: Networks of plausible inference Burlington, Massachusetts: Morgan kaufmann.

[ref76] Pearl J. (2000). Models, reasoning and inference. Cambridge, UK: Cambridge University Press, 19.

[ref77] Pearl J. (2009). Causal inference in statistics: an overview. Stat. Surv. 3, 96–146. doi: 10.1214/09-SS057

[ref78] Pearl J. (2011). Bayesian networks. UCLA: Department of Statistics, UCLA. Available at: https://escholarship.org/uc/item/53n4f34m

[ref79] Pearl J. (2012). “The causal foundations of structural equation modeling.” in Handbook of Structural Equation Modeling. ed. R. H. Hoyle (New York: Guilford), 68–91.

[ref80] Penrose R. (1994). Shadows of the mind 4 Oxford University Press Oxford.

[ref81] Penrose R. (1989). The emperor’s new mind: Concerning computers, minds, and the laws of physics American Association of Physics Teachers. New York: Oxford University Press.

[ref82] Pereyra N. A. (2020). “Review of logic and set theory.” in Real and Complex Numbers for Physicists. (Melville, New York: AIP Publishing LLC), 1–1.

[ref83] Pinna T. Edwards D. J. (2020). A systematic review of associations between Interoception, vagal tone, and emotional regulation: potential applications for mental health, wellbeing, psychological flexibility, and chronic conditions. Front. Psychol. 11:1792. doi: 10.3389/fpsyg.2020.01792 32849058 PMC7419655

[ref84] Pizlo Z. Li Y. Sawada T. (2014). Making a machine that sees like US: Oxford University Press, USA.

[ref85] Pollan M. (2015). The trip treatment. The New Yorker, 9.

[ref86] Prakash C. (2020). On invention of structure in the world: interfaces and conscious agents. Found. Sci. 25, 121–134. doi: 10.1007/s10699-019-09579-7

[ref87] Prakash C. Stephens K. D. Hoffman D. D. Singh M. Fields C. (2021). Fitness beats truth in the evolution of perception. Acta Biotheor. 69, 319–341. doi: 10.1007/s10441-020-09400-0, PMID: 33231784

[ref88] Schrödinger E. (1942). What is life?, Cambridge, Massachusetts: Cambridge University Press.

[ref89] Schwartenbeck P. FitzGerald T. Dolan R. Friston K. (2013). Exploration, novelty, surprise, and free energy minimization. Front. Psychol. 4:710. doi: 10.3389/fpsyg.2013.00710 24109469 PMC3791848

[ref90] Seth A. K. (2013). Interoceptive inference, emotion, and the embodied self. Trends Cogn. Sci. 17, 565–573. doi: 10.1016/j.tics.2013.09.007, PMID: 24126130

[ref91] Sheline Y. I. Barch D. M. Price J. L. Rundle M. M. Vaishnavi S. N. Snyder A. Z.. (2009). The default mode network and self-referential processes in depression. Proc. Natl. Acad. Sci. 106, 1942–1947. doi: 10.1073/pnas.0812686106, PMID: 19171889 PMC2631078

[ref93] Smith J. M. (1982). Evolution and the theory of games, Cambridge, Massachusetts: Cambridge universitypress.

[ref94] Smith P. Hayes S. C. (2022). An open-source relational network derivation script in R for modeling and visualizing complex behavior for scientists and practitioners. Front. Psychol. 13:914485. doi: 10.3389/fpsyg.2022.914485, PMID: 35783756 PMC9240703

[ref95] Smith J. Price G. R. (1973). The logic of animal conflict. Nature 246, 15–18. doi: 10.1038/246015a0

[ref96] Sörbom D. (1975). Detection of correlated errors in longitudinal data. Br. J. Math. Stat. Psychol. 28, 138–151. doi: 10.1111/j.2044-8317.1975.tb00558.x

[ref97] Sotres-Bayon F. Bush D. E. LeDoux J. E. (2004). Emotional perseveration: an update on prefrontal-amygdala interactions in fear extinction. Learn. Mem. 11, 525–535. doi: 10.1101/lm.79504, PMID: 15466303

[ref98] Zettle R. D. Hayes S. C. Barnes-Holmes D. Biglan A. (2016). The Wiley handbook of contextual behavioral science, New York: John Wiley & Sons.

[ref99] Zhu X. Wang X. Xiao J. Liao J. Zhong M. Wang W.. (2012). Evidence of a dissociation pattern in resting-state default mode network connectivity in first-episode, treatment-naive major depression patients. Biol. Psychiatry 71, 611–617. doi: 10.1016/j.biopsych.2011.10.035, PMID: 22177602

