# Supplementary material for: Functional contextual implementation of an evolutionary, entropy-based, and embodied free energy framework: Utilizing Lagrangian mechanics and evolutionary game theory’s truth vs. fitness test of the veridicality of phenomenological experience
Source: Front Psychol. 2023 Apr 11;14:1150743. doi: 10.3389/fpsyg.2023.1150743 (PMC10126492; doi:10.3389/fpsyg.2023.1150743)
Supplement: Supplementary file 1 [file Data_Sheet_1.PDF]

## Supplementary Material

### Supplementary 1

There is a likelihood value for each sensory stimulation given a world state, and this is given by $P(x_1 \mid w)$. These values are indicated in the first two columns of Table 1B, for instance, $P(x_1 \mid w_2)$ = ¾ (75%). Prior probabilities of world states $w$ are given in the third column, and the fourth column indicates the fitness associated with each world state. In this example, the world states contain a type of resource such as food, whereby $w_1$ corresponds to highly healthy but rare food, whilst $w_2$ and $w_3$ correspond to less healthy but more common food, and with $w_3$ being the least healthy. Observers are given two sensory state experiences $x_1$ and $x_2$, and they must choose between them.

In the case of a truth strategy observer, using Bayes' theorem, the truth estimate for $x_1$ is $w_2$ (see step 2) whilst for $x_2$ the estimate is $w_3$ (see step 3). To compute the truth estimate, Bayes' theorem must be used. First, however, it is important to note (particularly for step 4) that the set $W$ is considered, mathematically, a compact Borel space whose collection of measurable events is a σ-algebra denoted as $B$. $< W, B >$ has a prior probability measure $\mu$ on $B$. The uniform or Borel probability measure of $W$ is denoted as $dw$, and if this a priori measure is assumed, this satisfies $\mu(dw) = g(w)dw$. In this case, $g : W \rightarrow R^+$ is some non-negative measurable called the density of $\mu$ and satisfies $\int g(w)dw = 1$. This gives the structure of the world. So, to compute the truth estimate, the probability of each stimulation $\mathbb{P}(x_1)$ and $\mathbb{P}(x_2)$ must be determined from Bayes' theorem $P(w|x_0) = \frac{P(x_0|w) \cdot P(w)}{P(x_0)}$ (see equation 1 for full description) in the following way:

**Step 1.** First, $P(x_0)$ for sensory states $x_1$ and $x_2$ will be used as the denominator in Bayes' theorem in step 2.

$$\mathbb{P}(x_1) = (x_1 \mid w_1)\mu(w_1) + (x_1 \mid w_2)\mu(w_2) + (x_1 \mid w_3)\mu(w_3)$$

$= \frac{1}{4} \cdot \frac{1}{6} + \frac{3}{4} \cdot \frac{3}{6} + \frac{1}{4} \cdot \frac{3}{6} = \frac{13}{24}$

$\mathbb{P}(x_2) = (x_2 |\ w_1)\mu(w_1) + (x_2 |\ w_2)\mu(\ w_2) + \ (x_2 |\ w_3)\mu(\ w_3)$

$= \frac{3}{4} \cdot \frac{1}{6} + \frac{1}{4} \cdot \frac{3}{6} + \frac{3}{4} \cdot \frac{3}{6} = \frac{5}{8}$

**Step 2.** Then from the numerator of Bayes' theorem $P(x_0|w) \cdot P(w)$ posterior probabilities of the world states, given $x_1$ can be given and then divided by the denominator $P(x_1)$ value given in step 1:

$P(w_1 \mid x_1) = P(x_1 \mid w_1)) \cdot \frac{\mu(w_1)}{P(x_1)} = \frac{1}{4} \cdot \frac{1}{6} / \frac{13}{24} = \frac{1}{13} = 0.08$

$P(w_2 \mid x_1) = P(x_1 \mid w_2)) \cdot \frac{\mu(w_2)}{P(x_1)} = \frac{3}{4} \cdot \frac{3}{6} / \frac{13}{24} = \frac{9}{13} = 0.69$

$P(w_3 \mid x_1) = P(x_1 \mid w_3)) \cdot \frac{\mu(w_3)}{P(x_1)} = \frac{1}{4} \cdot \frac{3}{6} / \frac{13}{24} = \frac{3}{13} = 0.23$

These indicate that the maximum a posterior truth estimate for stimulus $x_1$ is $w_2$ (=0.69).

**Step 4.** Given a fitness function $f\colon W \to [0,\infty)$ that assigns to each state a non-negative fitness value, the expected fitness function of a perceptual state $x$ is $F(x) = \int f(w)\mathbb{P}(dw\ |x) = \int f(w)g(w|x)dw$. Using this expected fitness function, the expected-fitness values of the different sensory stimulations $x_1$ and $x_2$ are respectively:

$F(x_1) = \ P(w_1 \mid x_1)f(w_1) + \ P(w_2 \mid x_1)f(w_2) + \ P(w_3 \mid x_1)f(=\ w_3) = \frac{1}{13} \cdot 19 + \frac{9}{13} \cdot 5 + \frac{3}{13} \cdot 4 = \mathbf{5.85}$

$F(x_2) = p(w_1 \mid x_2)f(w_1) + \ P(w_2 \mid x_2)f(w_2) + \ P(w_3 \mid x_2)f(=\ w_3) = \frac{6}{35} \cdot 19 + \frac{6}{35} \cdot 5 + \frac{18}{35} \cdot 4 = \mathbf{6.17}$

As $x_2$ has a larger expected fitness than $x_1$, $x_2$ is selected.

**Supplementary 2**

In other simulations, greater complexity has been accounted for such as when using agent-based models that utilize Monte Carlo simulations. This is done by varying the number of territories thus shifting the boundaries, the number of resources per territory, and the correlations between resources (Mark, Marion, & Hoffman, 2010). In all of these cases, the

fitness (interface) only strategy has been shown to bring the truth perceptual strategy to extinction. In some cases, however, it has been suggested that by accounting for even greater complexity by adding another layer of real-world modeling in the form of simulating sudden environmental changes, then the truth perceptual strategy brings the fitness-only strategy to extinction (Charan, Gharibzadeh, & Firouzabadi, 2021). However, this approach assumes that the interface cannot adjust to sudden changes in the environment $w$, but this is unlikely to be the case. As the interface is mapped directly to fitness, its perceptual strategy is not concerned with the actual changes in the physical environment, instead it is only concerned with the change in fitness. Having to process all the new information using a Bayesian truth MAP would have additional cost to energy of the organism, and this information cost to the organism using the fitness-only strategy would be much lower (as the information content described by information theory is lower).

This can be shown in a simple way, through the same evolutional game theory approach used earlier (in Table 2). In the same simple example, where there are three world states, $W = \{w_1, w_2, w_3\}$ and two sensory state stimulations $X = \{x_1, x_2\}$, the only difference in this second turn of the game is that there has been a sudden environmental change that has flipped all of the values of fitness $f(w_j)$, prior probabilities $P(w_j)$, and the likelihood of world $w_j$ given some sensory state $x_1$ and $x_2$, $P(x \mid w_j)$ as shown in Table 3. For instance, the fitness values in the first game for worlds $w_1, w_2$, and $w_3$ were 19, 5, and 4 respectively, and in game two they were respectively 6, 5, and 21. These extreme changes simulate a sudden change in the environment.

In the example above, using the same mathematical approach as for Table 2, given a choice between$x_1$ and $x_2$, the truth strategy chooses $x_2$ = 0.69, whereas given a choice between $x_1$ and $x_2$, the fitness strategy chooses $x_1$ = 14.8. As 14.8 is a higher fitness value

than 0.69, fitness once again drives the truth strategy into extinction despite the sudden change in the environment.

**Supplementary 3**

The relational frame of distinction can be simply defined as the negation of equivalence, and this can be denoted as $\neg(A \sim B)$, or in another way $A \otimes B$, therefore $\neg(A \sim B) \equiv A \otimes B$. In set theory, this can be expressed as $A - B = \{ x \mid x \in A \; and \; x \notin B \}$.

To express a relational frame of similarity or sameness in set theory such that $A = B$ and $B = A$ this can be done in the following way (assuming all elements within the sets are the same): Since $A$ is equal to $B$, this can be represented as $A = B$, and this means that the set $A$ and set $B$ have the same elements. Also, as $B$ is equal to $A$, this can be represented as $B = A$, and this means that the set $B$ and set $A$ have the same elements. This can be represented through set theory as follows:

$A \cap B = A \cup B = \{x \mid x \; is \; an \; element \; that \; belongs \; to \; either \; A \; or \; B, or \; both\}$.

This means that the intersection of $A$ and $B$ is equal to the union of $A$ and $B$, and this is because all of the elements that belong to $A$ also belong to $B$, and all of the elements that belong to $B$ also belong to $A$. Additional propositions could be added if only some elements of the sets were the same and others were not.

A relational frame of hierarchy can be expressed using set theory by defining a set of elements, where each element belongs to one or more subsets, and each subset belongs to one or more supersets. The hierarchy is then defined by the relationships between these subsets and supersets, such as "is a subset of" or "is a superset of". In terms of logic, the hierarchy can be represented using propositional logic statements, where each element is represented by a propositional variable, and the relationships between subsets and supersets are represented by logical operators such as AND, OR, IMPLIES, etc. For example, if $A$ and $B$ are subsets of

$C$, such that $A = Alsation$, $B = Dog$, and $C = mammel$ then the statement "$A$ and $B$ are subsets of $C$" can be represented as $A \subseteq C \wedge B \subseteq C$ where $\subseteq$ is the subset symbol. More concretely $A \subseteq C$ iff $\forall\, x(x \in A \rightarrow x \in B)$ and $B \subseteq C$ iff $\forall\, x(x \in B \rightarrow x \in C)$. This means that for every element $x$ in $A$, it is also an element of $B$ and for every element $x$ in $B$, it is also an element of $C$. In other words, $A$ is a subset of $B$ and $B$ is a subset of $C$, indicating a hierarchical relationship between the sets, with $A$ at the lowest level, $B$ in the middle level, and $C$ at the highest level. In logic, the hierarchy of the statement (if $A$ then $B$) and (if $B$ then $C$) can be represented as $(A \rightarrow B) \wedge (B \rightarrow C)$, where $\rightarrow$ is a logical connective "implies".

Causal relational frames can be expressed as a relation symbol $R$ such as $aRb$ (where $R$ as a specific relational property is explicitly defined given some context) or as a function $f$ map to a set, such as $f(A) = B$ which means that some functional relation $A$ causes $B$. This can also be expressed as $f : A \rightarrow B$, which depicts a function, such that $A$ implies $B$. These functions are mathematical expressions and could include a transfer for function (ToF).

Deictic perspective-taking relational relations are complex and dynamic frames that relate the self within some context such as spatially (HERE vs. THERE), temporally (NOW vs. THEN), and interpersonally (I vs. YOU). These can be expressed in logic using quantifiers, for example, the deictic relation "I-YOU" can be represented using the first-order predicate logic quantifiers "for all" ($\forall$) "there exists" ($\exists$). For example, the statement "I am faster than YOU", can be represented as $\forall x(\text{person}(x) \rightarrow (\text{Fast}(x) \rightarrow \text{Fast}(I) \wedge \text{Slow(you)}))$. This can be read as: "For all $x$, if $x$ is a person and $x$ is fast, then $I$ am fast and you are slow." Similarly, the spatial deictic relation HERE-THERE can be represented in the same way using qualifiers "for all" and "there exists". For example, the following statement "Over THERE is a book on the table" can be represented as $\exists x(\text{Book}(x) \wedge \text{OnTable}(x))$. This reads as "There exists an $x$ such that $x$ is a book and $x$ is

on the table." These can also be expanded upon using set theory as shown with the other relational frames for more complex expressions.

In the case of a transformation of stimulus function (ToF), where for instance, you are afraid of snakes, and then you are told by a snake expert they live in the local woods, a relational frame coordinating snakes with the local woods form. With this coordination framing, the fear function from the snakes can then transfer to the woods (a ToF occurs). Again, a representation of this using logic and set theory can be made, whereby the set of all things you are afraid of, denoted here as $F = \{x \mid x\ is\ a\ snake\}$ and $F$ represents fear. Then as you frame snakes with the woods, this can be represented as a function typically donated as $f$, but here is denoted by $T$ to distinguish from other functions, whereby $T(x) = y$, and $x$ is a snake, whilst $y$ is the local woods. The transformation of the function of fear to the woods can then be represented as $T(F) = \{y \mid y\ is\ a\ local\ wood\ and\ \exists x(x\ is\ a\ snake \wedge T(x) = y)\}$. Explicitly, this translates into "the set of woods that you are afraid of is the set of all woods that are framed with a snake through the function $T$."

**Supplementary 4**

Some example python code connecting two nodes that represent snake and woods as these are framed in connection.

```
import networkx as nx

# Create an empty graph
G = nx.Graph()

# Add nodes representing elements in the community
G.add_node('snake')
G.add_node('wood')

# Add edges representing relationships between the nodes
G.add_edge('snake', 'wood')

# Use graph theory techniques to analyze the community
shortest_path = nx.shortest_path(G, 'snake', 'wood')
pagerank = nx.pagerank(G)

print(shortest_path)
print(pagerank)
```

**Supplementary 5**

Some example python code that represents the concept of a function, $T$, that transforms (ToF) the set of things you are afraid of, $F$, to the set of woods that you are no afraid of, $W$.

```
# Define the sets F and W
F = {'snake'}
W = set()

# Define the function T
def T(x):
  if x == 'snake':
    W.add('wood')

# Apply the function T to the set F
for x in F:
  T(x)

# Print the resulting set W
print(W)
```

**Supplementary 6**

Here, a larger graph needs to be defined such as graph $H$. The structure of the graph could be:

$H$({“$A$”, “$B$”,”$C$”,”$D$”,”$F$”,”$T$”,”x”,”y”,”Community 1”,”Community self”},{(“$A$”,”$B$”),(”$B$”,”$C$”), ("$C$","$D$"), ("$F$","$x$"), ("$x$"," $T$"),("$A$","$F$"), ("$D$","$T$"), ("$F$","$Community$ 1"),("$T$","$Communityy$ 1"), ("$Community$ 1, $Self$")}). In this example, nodes {"$F$","$T$","$x$","$y$"} and edges {("$F$", "$x$"), ("$x$", "$T$"), ("$T$", "$y$")} of G are included in the graph $H$ as “Community 1”. This community is then connected with the rest of the graph with edges ("$A$","$F$"),("$D$","$T$") and “Community Self”.

**Supplementary 7**

This concern relates to normative (large population) statistical approaches typically adopted in psychometric studies that make assumptions in order to examine consistencies among collections of individuals. Specifically, a growing number of researchers argue that this

normative (nomothetic) approach is mathematically not sensitive enough when assessing processes of change within individual people. They suggest that is difficult or impossible to use data collected from multiple subjects (inter-subject variability) to accurately model the variations with a single subject (intra-subject variability) in clinical behavioral science. They suggest that this is because the conditions required to do this properly are very restrictive and difficult to meet. This ergodic error has been shown to exist, in that its assumptions can only be made if one ignores the very many individual differences between individuals (P. C. Molenaar, 2013). It, therefore, becomes clear that precise inter-individual variation cannot be captured by current nomothetic approaches once psychological phenotypes are understood not to be ergodic in nature (P. C. Molenaar, 2008).

The reliability for scales across people in the form of cross-sectional data has been shown to be unreliable when attempting to inform scales across time for one individual such as when using time series data (Fuller-Tyszkiewicz et al., 2017; Hu et al., 2016). As such, inter-individual variation cannot accurately assess the contribution of given elements to phenotypic change. Because of these challenges, the current focus within recent developments within clinical psychology through PBT, has been to explore and conduct analysis at the individual, idiographic and idionomic level which are sufficiently capable at capturing such elements of inter-individual variation that lead to phenotypic change (Ciarrochi et al., 2022; Hayes & Hofmann, 2017, 2018; Hayes et al., 2021; Hofmann & Hayes, 2019). This highlights the need for more personalized individual-level assessment, particularly the case when modeling at the level of the relational frame, given this is based entirely on the very specific learning history and context of each individual.

This ergodic problem also extends to current psychiatric assessment models, such as identified in the current DSM model (American Psychiatric Association & Association, 2013) that highlight the need for protocols for syndromes, which again use a normative approach to

assume process-based change can occur at the individual level of intra-subject variability. This leads to problems of co-morbidity between categories of syndromes, which then leads to great difficulty in attempting to explain the clinical etiology and causal pathways between such syndromes. One study explored through a multi-level structural equation modeling the multi-path nature and problem of co-morbidity within these normative protocols for syndromes approaches (D. Edwards, 2022), and suggested more ideographically sensitive approaches.

**Supplementary 8**

These types of EMAs are particularly useful for studying changes in the individual over time (Dunton, 2017; Shiffman, Stone, & Hufford, 2008). These have been used in stand-alone studies as well as to compliment Randomized Controlled Trials (RCT) as follow-up studies (Perski et al., 2019) to gain a more in-depth analysis of the individual difference influences. Using an EMA this way ideographically could lead to a set of RFT-type time series assessment questions such as: “To what degree do you find yourself entangled with your thoughts about future or past events?”, whereby this assesses present moment feelings about how entangled the individual feels with their thoughts. “To what degree are you able to connect to present moment sensations in the here and now, rather than getting stuck thinking about the future or the past in this moment?”, whereby this assesses the individuals' feelings about how able they feel that can connect to the present moment. “To what degree do you spend worrying about the future in this moment?”, whereby this assesses future thinking. “To what degree do you spend thinking and feeling regretful, resentful, or pain about something in the past in this moment?” whereby this assesses thinking about the past. These are just some examples of ways to assess deictic frames, given an idiographic approach as they are presented in a way that asks about though and feelings in the present moment.

Machine learning approaches such as natural language processing (NLP) approaches could also screen more qualitative present moment descriptions, and then model specific relational frames identified within the text, such as expanding on recent work in relational framing AI (D. J. Edwards et al., 2022) with an NLP module.

**Supplementary 9**

Illustrating some simple python programming language code that use modules networkx and matplotlib to form a graph that uses an autoregressive VAR model.

```
import networkx as nx
import matplotlib.pyplot as plt

# Create a directed graph
G = nx.DiGraph()

# Add nodes for the variables in the VAR model
G.add_node("A")
G.add_node("B")
G.add_node("C")

# Add edges to represent the relationships between the variables
G.add_edge("A", "A", weight=2)
G.add_edge("A", "A", weight=1)
G.add_edge("B", "C", weight=1)

# Use the spring layout to position the nodes
pos = nx.spring_layout(G)

# Draw the graph
nx.draw(G, pos, with_labels=True)

# Show the plot
plt.show()
```

**Supplementary 10**

These strange loops which are thought to arise as unique properties of cognition are suggested to have a cyclic structure that goes through several levels of a hierarchical system,

where each level is linked to at least one other level through some types of relational connection. As an example of this, consider the following statement: “I am a liar”. If this is expressed as a logical statement and found to be true, then I must have been stating a truth, and if I am stating truth, then I cannot be a liar – hence the self-referential paradox. In a similar way, if “I am a liar” was expressed as a logical statement and found to be false, then I must have been lying about being a liar, therefore I cannot be a liar as I said a statement of truth – again hence the self-referential paradox. This also leads to the other types of paradoxes in self-referential statements such as referencing the system itself as in the following paradox, called the Liar Paradox: “This statement is false”.

In formal logical axioms of mathematics, this can at times be a problem. In the early 20 century, mathematicians were seeking a solid foundation for mathematics, mathematical facts or axioms that were both consistent and without contradiction to serve as the building blocks for all mathematical truths. Gödel (Gödel, 1931) used a modified version of this liar paradox example, to show that certain self-referential statements led to mathematical proofs of truth but were clearly false, and hence they were incomplete. Gödel modified the ‘liar paradox’ by stating instead “this sentence is unprovable”, and called the Gödel sentence G.

Gödel showed this paradoxical incompetence in self-referential logical systems formally in the following way: In a formal system $F$, and for every number $n$ and every formula $F(y)$ where $y$ is a free variable, $q(n, G(F))$, this can be defined as a relation $q$ between two numbers $n$, whereby $q$ takes the argument of a Gödel number for a formula (an arbitrary ascribed number for a formula) $G(F)$, and $q(n, G(F))$. These correspond to the statement “$n$ is not the Gödel number of a proof of $F(G(F))$”. Taking this one step further, any proof of $F(G(F))$ can be encoded by a Gödel number $n$ such that $q(n, G(F))$ does not hold true. If $q(n, G(F))$ holds true for all natural numbers of $n$ then there is no proof of $F(G(F))$. Therefore, $\forall$ for all instances of $y$, $\forall y \; q(y, G(F))$ is a formula about natural

numbers that correspond to "there is no proof of $F(G(F))$." If a formula is then defined $P(x) = \forall y\ q(y, x)$ where $x$ is a free variable, the formula $P$ has an ascribed Gödel number $G(P)$ as with all formulas in this exercise. If the free variable $x$ is then replaced with $G(F)$ then $P(G)F)) = \forall y\ q(y, G(F))$ also corresponds to "there is no proof of $F(G(F))$". Now consider the formula $P(G)P)) = \forall y\ q(y, G(P))$ where formula $F$ has been ascribed a Gödel value $P$, which corresponds to "there is no proof of $P(G)P))$". This brings the same paradoxical self-referential problem to formal axioms in logic as the simple Liar paradox i.e., it is a formula of the theory that relates to its own probability within the formal theory. As such, because of this self-reference, the formula $P(G)P))$ nor its negation $\neg P(G)P))$ is provable. If $P(G)P)) = \forall y\ q(y, G(P))$ were provable and let $n$ be the Gödel number of a proof $P(G)P))$, then the formula $\neg q(n, G(P))$ (the negation of the non-provable statement) is provable. However, proving both $\forall y\ q(y, G(P))$ and $\neg q(n, G(P))$ violates the consistency of the formal theory because of this self-reference, and hence it is concluded that $P(G)P))$ is not provable, and it is therefore concluded to be formally paradoxical, contradictory, and incomplete.

Hofstadter (Hofstadter, 1979, 2007) argues that a similar paradox occurs in the development of the psychological 'self' pointing to Gödel and the Liar paradox as evidence for this (as well as many other examples). He refers to humans' ability to use self-symbols and higher hierarchical levels of cognitive symbols that allow for deeper semantic meaning in understanding the paradoxical nature of Gödel's self-referential unprovable statements and other strange loops. Importantly, Hofstadter suggests that self-symbols arise as the individual interacts with their surroundings, such as perspective-taking about themselves (described in RFT as an I-NOW-HERE relation) and reflecting from other people's point of view (described in RFT as a YOU-NOW-THERE relation). The symbols which capture patterns from the environment are suggested to have emerged from Darwinian evolution in the form

of cognitive abilities such as categorization (including self-categorization or these could be deictic framing as described by RFT) that promote survival at a macro-level reality.

The self-symbols are suggested to grow with constant feedback loops, as the interaction with the environment increases over time. Hofstadter (Hofstadter, 1979, 2007) suggests that the level of hierarchical sophistication within the symbolic networks projects the level of reality that it attempts to mirror within the patterns given from within the environment. Therefore, the self-symbols project a reality about oneself based on the patterns from the environment it learns from (via reinforcement learning) within its hierarchical feedback loop. This is highly consistent with the RFT model which also suggests that complex networks are created via reinforcement and relational framing of hierarchy given patterns of contextual cues from the environment (Barnes-Holmes, Hayes, & Roche, 2001; Blackledge, 2003; Zettle, Hayes, Barnes-Holmes, & Biglan, 2016).

This could mean that self-symbols that project a negative reality about oneself may significantly and causally affect the behavior and thoughts of that individual in a negative way (possibly bring about depression, and perhaps destructive behaviors) consistent with the self-symbols. However, such self-referential thoughts or statements may be paradoxical, based on projected evaluation and not statements of truth. This is because the self-referential symbols about oneself are in a "strange" hierarchical feedback arrangement loop that has causal influence over the individual's agency and how they perceive and categorize the reality about themselves. This could be similar to the negative feedback loops described in PBT assessment work (Hofmann, Hayes, & Lorscheid, 2021). From this perspective, destructive self-referential propositions about oneself can be made (confirmation biases) such as "I am a loser", "no one likes me", "what is the point in trying, I will only fail", maybe incorrectly assumed to be true by the individual despite much counterfactual evidence which individual ignores. In ACT and RFT (Hayes, Strosahl, Bunting, Twohig, & Wilson, 2004;

Hayes, Strosahl, & Wilson, 1999, 2011), this type of self, is described as 'self-as-content', as is the self that is driven and entangled in these arbitrary self-symbols. This type of self may exist within the logical system of formal logical self-referential and paradoxical statements that describes it. This is distinct from an observer self (or self-as-context) which in ACT and RFT is described as the self that is not entangled by the self-referential system that describes it. It is this observer self or self as context that needs to be mathematically described outside and entirely unbounded from the formal logical system.

As a formal logical axioms account of the problem of self-reference strange loops, consider the following propositional statement: (1) "Bill is my enemy"; (2) "Bill is also his own worst enemy"; (3) "The enemy of my enemy is my friend"; (4) as Bill is the enemy of my enemy, he must be my friend." This is clearly paradoxical in nature, but it can be proved within formal logic to be true. For example:

Let $E(x, y)$ represent the statement "$x$ is an enemy of $y$", and $FW(x)$ be the statement "$x$ is his own worst enemy", and $F(x, y)$ be the stamen "$x$ is a friend of $y$". The argument expressed in propositional statements one to four can be expressed as a theorem within a formal mathematical system, using the following mathematical notation:

Theorem: $\forall x, y, z(E(x, y) \land E(y, z) \rightarrow F(x, z))$, which states that for any individual $x$, $y$ and $z$ if $x$ is an enemy of $y$ and $y$ is an enemy of $z$ (this expresses that Bill $x$ is his own worst enemy $z$), then $x$ must be a friend of $z$ (who is Bill expressed as an enemy of himself).

To show that this theorem is true, a proof by contradiction can be provided in 11 steps, such as:

1. Assume that $\forall x, y, z(E(x, y) \land E(y, z) \rightarrow F(x, z))$ is false.
2. Then there exists $x, y, z$ such that $E(x, y) \land E(y, z)$ and $\neg F(x, z))$ .
3. From step 2, we know that $x$ is an enemy of $y$, and $y$ is the enemy of $z$.
4. From step 3, we know that $x$ is not a friend of $z$.

5. From step 4, we know that $x$ and $z$ are not friends, and since they are not friends, they must be enemies.
6. From step 5, we know that $x$ is an enemy of $z$.
7. From step 6, we know that $E(x, y)$.
8. From steps 3 and 7, we know that $y$ is an enemy of $x$ and $y$ is an enemy of $z$.
9. From step 8, we have $E(x, y) \wedge E(y, z)$.
10. From steps 9 and 1, we have $F(x, z)$ which contradicts 2.
11. Therefore, the assumption made in step 1 must be false, and thus $\forall x, y, z(E(x,y) \wedge E(y,z) \rightarrow F(x,z))$ is proved true.

This proof shows that the theorem $\forall x, y, z(E(x,y) \wedge E(y,z) \rightarrow F(x,z))$ (Bill is both your friend and your enemy) holds true within a formal mathematical logical system, and it's a valid and provable mathematical statement despite it being paradoxical and an example of a strange self-referential strange loop expressed within formal logical mathematics.

For this reason, some aspects of self-such as the observer self (the conscious aspect of self that observes the individual's experience) needs to be described mathematically outside of the formal system that attempts to describe it at least partially prevent such self-referential strange loops. One potential way to explain this observer self outside of logical systems is to use a different mathematical framework, such as a Markov Kernel. A Markov kernel is a mathematical framework used in the field of machine learning and optimal control theory to model the probability of transitions between different dynamic states within the system. This may be one way to better understand and model this complex construct of the conscious observer self.

**Supplementary Material 11**

In accordance with information theory, the representational contents of conscious experience $X$ and conscious action $G$ are assumed to be encoded in bits of information. $X$ and $G$ are also assumed to encode the same number of bits (the same resolution) to the inputs and outputs of world state $W$. Decisions $D$ can be assumed to operate in discrete steps, as previous instances of $G$, $D$ maps a fully encoded element of $X$ to a fully encoded element of $G$. The smallest amount of information of either $X$ or $G$ is one bit, and therefore the smallest amount of information of an action of $D$ is a one-bit to one-bit mapping.

The CA thesis suggests that a conscious process, such as conscious recognition (e.g., contextual functional cue), inference (e.g., derived relation), or choice should be representable through the action of a Markov kernel. It suggests that any formal representation of conscious experience must be represented through the interaction of the kernels $P - D - A$ cycle given some formal properties (Koenderink, 2014). As such, the observer self is described as a process of conscious experiences that consciously observes experience transmitted through the evolutionary defined interface (based on fitness). As previously justified through the 'fitness beats truth' theorems, that the objects and casual relations within conscious experience $X$ should not be assumed to be homomorphic to the elements and relations within the extrinsic world state $W$. This is important when using a functional contextual approach, as functional continual cues are complex, can change through relational framing (e.g., such as through a transformation of stimulus function), and are often hidden from the conscious perceiver.

Cognition and relational frames of RFT, including basic reinforcing properties can be applied at the interface level and represented through the interaction of the kernels $P - D - A$ cycle of the CA. This is done through the interplay of intrinsic and extrinsic perspectives of CA (the observer self). Extrinsic and intrinsic perspectives of CA can contrast, such that the extrinsic perspective relates to the theorists' perspective of elements $W$, $A$, and $P$, whilst the

intrinsic perspective of the CA is the conscious experience itself, i.e., the CA is an observer (the conscious observer perceptive – i.e., the observer self). The intrinsic CA phenomenological perspective can be formulated through the concept of a "reduced CA" (RCA), which is a 4-tuple $[(X, \mathcal{X}), (G, \mathcal{G}), D, t]$. It is this RCA combined with extrinsic perspective elements $W$, $A$, and $P$, that makeup CA. The RCA is free to choose which conscious actions $G$ they would like to take in response to some conscious experience $x \epsilon X$ when embedded and interacting with $W$, and represented by kernel $D$. The RCA's $A$ on $W$ is in part determined by the structure of $W$, and likewise, the $P$ is in part determined by the structure of the RCA (i.e., the structure of the observer self).

From this, it can be assumed through ITP that the RCA's knowledge of $W$ is formed entirely through conscious experience $X$. The RCA's entire conscious experience of $W$ at time $t$ can be given by the elements of $X$ that are selected by $P$ at any given time $t$. Through these assumptions, it is clear that ITP does not allow the RCA any independent access to the ontology of $W$, and therefore must be assumed to be a-ontological, and this is consistent with the a-ontological position of functional contextualism. As such, the RCA cannot access their own or others' $P, D,$ and $A$ kernel definitions, so have no way of knowing whether they are homomorphisms of the real world. An RCA only knows what appears in their experience of $X$ and has no other knowledge about the real world for itself or other RCAs (including other RCAs $X$ and $G$). If any structure is attributed to $W$, then this can only be hypothetical, and made from inferences (e.g., derived relations) of knowledge about the world experienced from $X$.

**Supplementary 12**

Interoceptive signals, that signal to the brain from the body to form embodied cognition originate from afferent neurons which signal to the brain interoceptive signals from the body via small-diameter unmyelinated C and myelinated Aδ primary afferent fibers. These fibers innervate all bodily tissue and terminate monosynaptically in lamina I and II neurons of the spinal and trigeminal dorsal horns (Craig, 2002; Panneton, 1991), and their outputted electrical signals then travel through the posterior grey column of the spinal cord to hypothalamus, anterior insular and cingulate cortices (Pollatos, Gramann, & Schandry, 2007).

The signals enter an integration phase, whereby they are then organized into primary emotional and motivational centers of the limbic system, the anterior insula cortex (AIC), and cingulate cortices of the homeostatic sensorimotor cortex. This integrative system is activated during all emotional and motivational behavior (Craig, 2014; Murphy, Nimmo-Smith, & Lawrence, 2003), so have an important role to play in terms of behavioral, regulatory, and even ER (Pinna & Edwards, 2020). At this stage, it has been proposed that these centers then develop and define a meta-representation of the self which allows for finely-tuned regulatory responses to be formulated (Damasio & Carvalho, 2013).

Damasio (Damasio, 2003; Damasio & Carvalho, 2013) who developed the somatic marker hypothesis of consciousness (which is the assumption that self-aware consciousness emerges from an image of the homeostatic state of the body), suggested that the interoceptive pathway of the right insular maybe involved with neuro basis of the conscious representation of the self. This assumption, along with suggestions made by Critchely et al. (Critchley, 2003) who through reviewing the imaging literature of emotion, suggested that subjective emotion and the role of interoception and the anterior insular still needed further modeling efforts to incorporate these, are interesting. They perhaps fit well with the suggestions made by LeDoux and colleague, where perhaps the meta-representation of self as produced by the interoceptive system of the insular cortex, maybe some form from a pre-representation before

a more complete conscious representation of 'self' emerges which involve higher levels of cognition within emotional processing in the frontal neocortex. (LeDoux, 2000, 2020; LeDoux & Brown, 2017). LeDoux's suggestion that conscious perceptual and emotional states depend on antecedent non-conscious states which include schemas, memories, and mental models (meta-cognition) may therefore include the interoceptive states as described by Damasio. Complex, hierarchical relational frames that relate deictics of self and other, may be involved in this frontal cortical area. This could be related to the hierarchical patterns of mind that were supposed to be important for self-referential thought as suggested by Hofstadter (Hofstadter, 1979, 2007).

The neurovisceral integration model (NIM) (Thayer & Friedman, 2002, 2004; Thayer & Lane, 2000) suggests that the anatomical network of the forebrain, brainstem, spinal cord and the central autonomic network (CAN) are delineated, and the integration of sensory-visceral, emotional, and cognitive information, as well as the regulatory actions as a result of this, is explained (Benarroch, 1993). Integrative processes involved such as the parabrachial nucleus, the nucleus solitarius, and medullary reticular formation of the brain stem (Thayer & Lane, 2000) for reflex control within the vagal nerve pathway, which project to areas such as the hypothalamus, and which should be noted. Central to the way in which emotional regulation, interoception, and heart rate variability are connected, seem to largely be via a feedback loop of brain circuitry which includes the limbic system; such as the amygdala (for fear processing), hypothalamus (integrating endocrine inputs), hippocampus (for Pavlovian associative memories), and thalamus (as a regulatory relay); the basal ganglia (for operant conditioning of rewards); the insular cortex (for interoceptive, as well as vagal properties and meta representation of self), along with the anterior cingulate cortex (ACC); and the prefrontal cortex (for higher-order cognition, emotional experience, and representation of self).

Evidence for the looping effects of this axis is given in several clinical studies which complement the many non-clinical studies already mentioned such as the NIM (Thayer & Friedman, 2002, 2004; Thayer & Lane, 2000), Polyvagal theory (Porges, 2003, 2007, 2018), interoceptive pathway (Craig, 2002, 2003; Strigo & Craig, 2016) but also other evidence such as the basal ganglia being involved in the reward system of operant reinforcement, through a thalamus-hippocampus connection (Aggleton et al., 2010); hippocampus-amygdala (Phillips & LeDoux, 1992; Sutherland & McDonald, 1990); hippocampus-amygdala-prefrontal cortex (McEwen, Nasca, & Gray, 2016); and hypothalamus-amygdala-prefrontal cortex (Buijs & Van Eden, 2000; Lundy Jr & Norgren, 2004).

From the clinical evidence, such connectivity has been identified within studies that relate to post-traumatic stress disorder (PTSD), which is characterized by altered and negative emotional responses as well as associated behavioral problems such as poor sleep, restlessness, hypervigilance, anhedonia, and social withdrawal. Amygdala-insula connectivity and activity have been found to be largely responsible for these altered emotional and behavioral states (Rabinak et al., 2011). In addition to this, there is clear evidence that suggests that the anterior cingulate cortex (ACC) connects and relays information to both the limbic system and the prefrontal cortex (Stevens, Hurley, & Taber, 2011). There is also evidence of bilateral insula-ACC connectivity (White, Joseph, Francis, & Liddle, 2010), where it was found that there was heightened amygdala responsivity in PTSD suffers when the individuals were stressed (i.e., symptomatic states) and exposed to trauma-related and affective situations. Crucially, it was found that prefrontal cortex responsivity was inversely associated with stress symptom severity, i.e., higher activation of the prefrontal cortex led to lower activity of the amygdala and associated stressful symptoms (Shin, Rauch, & Pitman, 2006). This may suggest that the prefrontal cortex has some inhibitory control over some aspects of the limbic system such as the amygdala.

**Supplementary 13**

Embodied cognition and self, have emerged in the cognitive science literature (Hohwy, 2020), and conceptualize the brain as a predictive machine (the Bayesian Brain hypothesis), which includes the idea of predictive coding, the free energy principle, and active inference (Ramstead, Kirchhoff, & Friston, 2020). The free energy principle (FEP) is widely considered a unifying theory that aims to explain the brain and the dynamics of all living organisms (Kirchhoff, 2018). The theory suggests that living, adaptive, self-organizing systems avoid disorder through dispersion by random fluctuations and attempt to remain in thermodynamic non-equilibrium steady-states by restricting themselves to a limited number of states through the minimization of free energy (prediction error) (Friston, 2009, 2019; Hipolito, 2019; Limanowski & Friston, 2020). A simpler definition of free energy can be stated as the difference between the system's predicted state and their actual state, whereby this difference is termed the prediction error, so that minimizing prediction error is functionally the same as minimizing free energy (Bohlen, Shaw, Cerritelli, & Esteves, 2021). Minimizing free energy can also be understood and explained through the avoidance of surprise and to minimize entropy (uncertainty) within the system (Friston, 2010).

Predictive coding deals with the management of entropy, and some of these ideas emerged decades ago, when the physicist Edwin Schrödinger (Schrödinger, 1942) in his seminal book, *What Is Life?*, argued that living adaptive systems survive by reducing their internal entropy, whilst increasing the entropy in their external environment. Schrodinger (Schrödinger, 1944) observed that living systems were unique among natural systems as they had the ability to self-organize over time, and therefore resisted the second law of thermodynamics which states that the entropy of an isolated system left to spontaneous

evolution cannot decrease – i.e., entropy must increase over time in these systems. This inspired a new center of inquiry called evolutionary systems theory (EST) which is related to complexity theory, and which explains dynamic, evolving systems, and the reciprocal relation between general evolutionary selection and self-organizing behavior (Badcock, 2012; Depew & Weber, 1996; Kauffman, 1993). This relates to the EEMM, in that behavioral selection and retention at the different dimensions and levels may be at a higher level of dynamical system analysis, maybe a form of entropy-reducing system.

Entropy is a concept derived from thermodynamics and information theory describing the amount of disorder or uncertainty within a system (Hirsh, Mar, & Peterson, 2012).
In accordance with the second law of thermodynamics, the total level of entropy within the universe will always increase, however, living (self-organizing) systems can reduce the entropy found within their biological systems through the consumption of energy from the external environment, and use it to maintain order within their system, and by displacing entropy in the environment. This works in line with dynamical systems theory, whereby the entropy-reduction framework has been extended to account for biological organisms as dissipative systems (Prigogine & Stengers, 1997). This account suggests that an organism must dissipate its entropy into the external environment in order to survive. In situations whereby the environment changes and produces more entropy for the organism within that environment, this challenges the structural (self-organization) coherence of the organism, and the organism must then adjust its pattern of self-organization to reduce the internal entropy and dissipate this within the environment. So, in dynamical systems theory, this refers to a biological organism as an information system, that must self-organize appropriately in order to manage its internal entropy in order to survive (Kauffman, 1993). Those who do not, are destroyed, and in evolutionary theory, this implies that the organism becomes extinct. In responding to increased external entropy, complex systems tend to return to a number of

smaller, stable entropy states called attractors (Grassberger & Procaccia, 1983), as the more complex systems cannot provide stable entropy management.

In addition to the study of an adaptive system's structural organization, the frameworks of entropy and self-organizing adaptive systems have been applied to the study of psychological phenomena (Barton, 1994; Carver & Scheier, 2002; Hollis, Kloos, & Van Orden, 2009; Vallacher, Read, & Nowak, 2002). This is because psychological phenomena can be regarded as a set of complex systems, and the information theory frameworks of entropy and self-organization can therefore be useful here. Examples of these include observations of self-organizing dynamics in cognitive processes of problem-solving (Stephen, Boncoddo, Magnuson, & Dixon, 2009; Stephen, Dixon, & Isenhower, 2009). In these studies, the researchers made two main observations: (1) that an increase in entropy of problem-solving behavior occurs when the initial strategy to solve a problem becomes ineffective. This increase in entropy was quantifiably measured by the increasing irregularity and unpredictability of the participant's responding to the problem as they attempted to solve it. (2) The increase in behavioral entropy preceded the subsequent changes in behavior to solve the problem, whereby more predictable, stable, and low entropy behavioral patterns were preferred and selected. This suggests that the cognitive-behavioral system seems to obey the same principled laws of internal entropy minimization as other dissipative self-organizing systems. So, from an evolutionary perspective, the system will select self-organizing behavior which minimizes internal entropy when it is confronted with environmental challenges of high entropy, in order to adapt, or the system will be overwhelmed, deteriorate, and fail to adapt, thus ultimately leading to its own extinction.

Similar entropy interpretive frameworks have been applied to neural activity which underly cognitive phenomena. As such, several quantitative entropy measures have been developed for the neural substrate (Borst & Theunissen, 1999; Nemenman, Bialek, & Van

Steveninck, 2004; Paninski, 2003; Pereda, Quiroga, & Bhattacharya, 2005; Strong, Koberle, Van Steveninck, & Bialek, 1998; Tononi, Sporns, & Edelman, 1994). Perhaps the most prominent of these theories is that provided by Friston and colleagues (Friston, 2010, 2013; Friston, Kilner, & Harrison, 2006; Friston, Parr, & de Vries, 2017) whereby they suggest that the brain tries to reduce internal neuronal entropy in order to support cognitive and behavioral adaptation, and by generating more adaptive (predictive) representations of the environment. Here, the brain, within a dissipative systems context, adjusts its structural organization (reducing entropy in the form of predictive error) as it acts as a self-organizing system, and adjusts to the changes in reinforcing contingencies and entropy within the environment (Friston, 2010; Kelso, 1995).

Evolution, biology, cognitive, and behavioral influences can be explored through an adaptive framework that fits well with EEMM. The role of entropy within a thermodynamical perspective or self-organizing, adaptive agents is to explain a wide range of cognitive, behavioral, and evolutionary phenomena. From a psychological perspective, when confronted with some environmental situation, the organism (here, an individual) is presented with an array of perceptual and behavioral affordances which reflect the combination of incoming sensory information from the environment with the organism's probability for responding cognitively and behaviorally (Cisek, 2007; Cisek & Kalaska, 2010; Gibson, 2014; Warren, 2006; Zhang & Patel, 2006). So, these affordances are assumed to direct the possible behavioral responses which can be implemented given some environmental context (Gibson, 2014). Perceptions relate to the interpretation of sensory information in accordance with expectations (predictions), motives (goals and values), and past experience (prior reinforcement and memories). An interesting entropy model of uncertainty (EMU) (Hirsh et al., 2012) conceptualizes perceptual and behavioral affordances as probability distributions. Within this EMU model (Hirsh et al., 2012), Shannon's entropy (Shannon, 1948) formula is

utilized to calculate the entropy associated with a given perceptual or behavioral experience. This can be given by the negative sum of the log probabilities of each possible perceptual or behavioral outcome, and is denoted as:

$$H(X) = -\sum_{i=1}^{n} p(x_i)\, log_2 p(x_i)$$

Where entropy $H$ of variable $X$ (in this case the perceptual or behavioral outcome), whereby possible outcomes $x_1, \dots, x_n$, which have the probability of occurrence $p(x_1), \dots, p(x_n)$ within some probability distribution, and represented as a function of weighted neural inputs for a possibility of a perceptual or behavioral event (e.g., $x_1$), in that moment, and given a specific environmental context. Here, the formula indicates probability distributions whereby one or more perceptual or behavioral outcomes are more likely to result than others and should reflect lower entropy levels, i.e., more certainty and predictability about the outcome of a particular perception or behavior given some environmental stimuli (or reinforcing contingencies). In contrast to this, Shannon's entropy equation suggests that probability distributions that are flatter, in which no perceptual or behavioral outcome is likely favored (in selection), then this should result in higher entropy, i.e., less certainty about an outcome, given some environmental context. Here, behaviors embedded in goals and values can be shown to reduce overall entropy over the longer term, so this seems ACT consistent.

**Supplementary 14**

The Helmholtz decomposition is particularly useful for exploring the stability and convergence in coupled dynamical systems. Here, it suggests that any sufficiently smooth vector field **F** which outputs continuous derivatives, can be decomposed into irrational (curl-free) and solenoidal (divergence-free) vector field. As the irrational vector field has only scalar potential, and the solenoidal vector field has only a vector potential, a vector field can

therefore be expressed as follows here $\nabla \Phi$ and $\nabla \times \mathbf{A}$ is the irrational and solenoidal vector fields respectively:

$$\mathbf{F} = -\nabla \Phi \nabla \times \mathrm{A}$$

Within dynamical systems theory, Lyapunov functions have been used extensively to model the stability of fixed points of dynamical systems (Liapunov, 2016; Lyapunov, 1992). They are generally defined for smooth systems in conditions:

(a) $L(x^*) = 0,$ and $L(x) > 0$ if $x \neq x^*$

(b) $\dot{L}(x) = \frac{dL}{dt}|_x\ 0,$ for all $x \epsilon O,$

Where $O \subseteq \mathbb{R}$ is an open set containing all states of $x$, where $\subseteq$ representants a subset. So, to summarize, in any nonequilibrium steady state dynamical system, the flow can be expressed as a scalar potential or Lyapunov function $\psi(x) = L(x)$ where the flow can always be decomposed into a gradient flow, and this minimizes the potential and a solenoidal component that flows on the iso-contours of the potential.

Finally, a mathematical term must express the association of the potential or Lyapunov function with variational free energy. Variational free energy is a function of internal states, that allows for the characterization of system dynamics in terms of Bayesian inference and implicit generative models. It unpacks the non-equilibrium steady-state flow of internal, external, and blanket states. Under this partition, internal and active states minimize variational free energy (instead of the thermodynamic potential or Lyapunov function). Variational free energy is defined in terms of a generative model and implicit posterior beliefs encoded within internal states. So, the minimization of variational free energy gives an interpretation of self-organization in terms of belief updating (the generative model

updates) according to the Bayes rule. This allows for the specification of the resulting non-equilibrium steady state in terms of a generative model.

The next step is to define the dynamics within a setting of generalized coordinates of motion and density dynamics as described by the Fokker-Plank equation. For generalized flow, this description of dynamics in generalized coordinates of motion is denoted with a tilde, where $\tilde{x}$ is defined as:

$$\tilde{x} = (x, \dot{x}, \ddot{x}, \dots)$$

This augments a state with its velocity, acceleration, etc. Generalized coordinates of motion will eventually be used to parameterize a posterior density over the general motion of external states which are hidden behind the Markov blanket. These general coordinates allow for the accommodation of temporal correlations in random fluctuations. When assuming a smooth dynamical system, subject to random fluctuations, the motion of states can be described by the Langevin equation, and denoted as:

$$\dot{\tilde{x}} = f(\tilde{x}) + \widetilde{\omega}$$

Where $f(\tilde{x})$ is the generalized flow or time evolution of states parameterized by the forces acting on the states, and $\widetilde{\omega}$ are the random fluctuations under the Wiener assumptions – i.e., the flow of states follows a process of independent, Gaussian increments that follow a continuous path. The evolution of the probability density $p(\tilde{x})$ through the Fokker-Plank equation can be obtained by the Langevin equation, using the conversion of probability mass:

$$\dot{p}(\tilde{x}) = \nabla \cdot \left[\dot{\tilde{x}} p(\tilde{x})\right] = 0$$

Where $\dot{\tilde{x}}p(\tilde{x})$ describes the probability current, and turns the Fokker-Plank equation into a continuity equation, which can be denoted as:

$$\dot{p}(\tilde{x}) = \nabla \cdot \Gamma \nabla p - \nabla \cdot (f(x)p)$$

A partial differential equation that describes the time evolution of the probability density $p(\tilde{x})$ under dissipative and conservative forces. The density dynamics, at a non-equilibrium steady state, is the solution to the Fokker-Plank equation, and is denoted as:

$$L(\tilde{x}) = -\, ln\, p(\tilde{x})$$

Such that $\nabla p = -p \nabla L$ and $\dot{p} = 0$. Then, utilizing the Helmholtz decomposition the steady-state flow can now be expressed in terms of a divergent-free component and a curl-free decent on a scalar Lyapunov function $L(\tilde{x})$, to obtain the following:

$$\dot{f}(\tilde{x}) = (Q - \Gamma) \nabla L(\tilde{x})$$

This is the solution at the non-equilibrium steady-state, and it is now possible to see that the Lyapunov function $L(\tilde{x})$ is the negative log probability of finding the system in any generalized state $L(\tilde{x}) = -\ln p(\tilde{x})$. This is also known as the self-information or surprise (surprisal) in information theory, and in Bayesian statistics, it is known as negative log evidence.

In addition to surprisal, goal-directed behavior (anchored in values) of the system needs to be defined mathematically. Here, the principle of least action, from physics is

relevant to describe how a self-organizing system works toward an invariant outcome despite various environmental changes, in the form of goal-directed behavior (or action). From this principle of least action, paths of behavioral least action can be predicted, whereby a path could be considered a flow channel for finding the least average action in situations of goal-directed behavior (e.g., gathering food).

Open dynamical systems prefer states of least action or in other words, the most efficient state. These dissipative random dynamical systems (Arnold, 1995; Crauel & Flandoli, 1994) do not minimize action for each element of the system but instead do so on average over an ensemble of elements (G. Georgiev & Georgiev, 2002; G. Y. Georgiev & Chatterjee, 2016; G. Y. Georgiev, Chatterjee, & Iannacchione, 2017; G. Y. Georgiev et al., 2015). As self-organizing systems are not conservative, they are inherently dissipative. As such, action is reduced by obstructive-constraint minimization for each event within the system and self-organized, which forms a flow structure and could be understood as a dissipative structure (England, 2015; Evans & Searles, 2002; Prigogine, 1978). Though the Lyapunov function of a physical system is utilized to establish the stability of a fixed point in a dynamic system, physicists commonly use the Lagrangian to solve the trajectory of a system's states. For a conservative system, the Lagrangian can be denoted as:

$$L = T - V,$$

Where $L$ denotes the Lagrangian, $V$ is the potential energy of a system and defined by the constraints of the system, $T$ is the kinetic energy of the particles which constitute the system such as the neurons. The trajectory of states in generalized coordinates $(t, \tilde{x}(t), \dot{\tilde{x}}(t))$ are given via the Euler-Lagrange question, which is bound by the principle of least action to be functions, and in the following is stationary (has extrema):

$$S(\tilde{x}) = \int_{t_1}^{t_2} L(t, \tilde{x}\ (t), \dot{\tilde{x}}\ (t))\mathrm{d}t.$$

$S$ integrates the Lagrangian of general states for boundary conditions defined for initial and final time points $t_1$ and $t_2$. The most likely path between two points can be obtained when the functional derivative is zero, i.e., $\delta S = 0$, which is the Hamilton's principle. The equations of motion are then derived from the Euler-Lagrange equations, which give the solution to the principle of least action, and are denoted as:

$$\frac{\mathrm{d}t}{\mathrm{d}t}\frac{\partial L}{\partial \dot{\tilde{x}}} - \frac{\partial L}{\partial \tilde{x}_i} = 0 \text{ for } i = 1.2, \dots, n$$

Where $\tilde{x}_i$ are the generalized coordinates and $\dot{\tilde{x}}_i$ the generalized velocities. The equation has additional dissipate terms for dissipative systems. The dissipative function depends on the square of the velocity, denoted as:

$$F = \frac{1}{2}\ k\dot{\tilde{x}}^2$$

Which converts the Euler-Lagrange equation into:

$$\frac{\mathrm{d}t}{\mathrm{d}t}\left(\frac{\partial L}{\partial \dot{\tilde{x}}_i}\right) - \frac{\partial L}{\partial \tilde{x}_i} + \frac{\partial F}{\partial \dot{\tilde{x}}_i} = 0$$

Here, the constraints to the motion of the agents in the system can be given by the Lagrangian multipliers:

$$\delta \int_{t1}^{t2} [L(t, \tilde{x}(t), \dot{\tilde{x}}(t)) + \sum_k \lambda_k(t) g_k(t, \tilde{x}(t))] dt = 0$$

Where $\lambda_k$ are the Lagrangian multipliers, and $g_k$ are the constraints (Arfken & Weber, 1999). The solutions for this are the constrained Lagrangian equations of motion., denoted as:

$$\frac{\mathrm{d}t}{\mathrm{d}t}\left(\frac{\partial L}{\partial \tilde{x}_i}\right) - \frac{\partial L}{\partial \tilde{x}_i} + \frac{\partial F}{\partial \tilde{x}_i} = \sum_k \lambda_k \frac{\partial L}{\partial \tilde{x}_i}$$

Random noise can also be included in the equation, which is important for biological systems such as neuron functioning (El Kaabouchi & Wang, 2015). The functional $S$ is the action of the system as the Lagrangian describes trajectories of particles under force. Therefore, the variational principle is applied to the action of a system and referred to as the least action principle. For larger biological systems, it is necessary to apply the action of an assemble of systems of particles when applying the least action principle.

This is reflected in the solution $L(\tilde{x}) = -\ln p(\tilde{x})$ to the Fokker-Plank equation, which means the action is the path integral of the marginal likelihood or self-information for any system or model $m$:

$$S = \int_{t1}^{t2} L(\tilde{x}(t))\, \mathrm{d}t = \int_{t1}^{t2} \ln p(\tilde{x}|m) \mathrm{d}t$$

**Supplementary Material 15**

$$\mu \perp \eta | b \Leftrightarrow p(\mu, \eta \,| b) = p(\mu | b) p(\eta | b)$$

This emphasizes a dynamical setting (Friston, Da Costa, & Parr, 2021), whereby the rate of change of each component within a Markov blanket. It suggests that the joint

probability $p$ between internal states $\mu$ and external states $\eta$ mediated by blanket states $b$ through the component $p(\mu, \eta \mid b)$, whereby $\mu$ states are independent $\perp$ from $\eta$ states belonging | to the blanket $b$, and is expressed as $\mu \perp \eta | b$. $\Leftrightarrow$ represents biconditional logical connective (or equivalence) between two statements (i.e., assumes both statements are true), where if $\mu \perp \eta | b$ is true then $p(\mu, \eta \mid b)$ must be true. This equation, in a dynamical setting (Friston et al., 2021), this means that the rate of change for internal $\dot{\mu}$, external $\dot{\eta}$, active $\dot{a}$, and sensory $\dot{s}$ states can only depend on two other states in accordance with this equation. The rate of change which preserves the conditional independence within this dynamical system can be denoted in below equation, for each component state between $b$ and $\eta$:

$$\dot{\mu} = f_{\mu}(\mu, s, a)$$

$$\dot{a} = f_{a}(\mu, s, a)$$

$$\dot{\eta} = f_{\eta}(\eta, s, a)$$

$$\dot{s} = f_{s}(\eta, s, a)$$

Here, $s, a$ comprise $b$, and the flow $f$ of a state such as an internal state $f_{\mu}$ is conditionally dependent on the structure of $b$. Equation 2 shows mathematically (like Figure 1 showed schematically), that the flows of internal $f_{\mu}$ and external states $f_{\eta}$ do not depend on one another. The Markov blanket refers to the structure of exchange between the organism and its environment (Friston, 2013; Kirchhoff, 2018; Parr & Friston, 2018), and specifically describes the self-organization across spatial and temporal scales (Hipolito, 2019; Palacios, Razi, Parr, Kirchhoff, & Friston, 2017; Ramstead et al., 2020). Within this context, the variable of interest can be associated with the internal sates $\mu$ of $b$ whereby the parents of $\mu$ which are the sensory states $s$ mediate the influence of eternal states $\eta$ and with the internal states $\mu$.

# References


Aggleton, J. P., O'Mara, S. M., Vann, S. D., Wright, N. F., Tsanov, M., & Erichsen, J. T. (2010). Hippocampal–anterior thalamic pathways for memory: uncovering a network of direct and indirect actions. *European Journal of Neuroscience, 31*(12), 2292-2307.

Arfken, G. B., & Weber, H. J. (1999). Mathematical methods for physicists. In: American Association of Physics Teachers.

Arnold, L. (1995). Random dynamical systems. In *Dynamical systems* (pp. 1-43): Springer.

Badcock, P. B. (2012). Evolutionary systems theory: A unifying meta-theory of psychological science. *Review of general psychology, 16*(1), 10-23.

Barnes-Holmes, D., Hayes, S. C., & Roche, B. (2001). Relational frame theory: A post-Skinnerian account of human language and cognition.

Barton, S. (1994). Chaos, self-organization, and psychology. *American Psychologist, 49*(1), 5.

Benarroch. (1993). *The central autonomic network: functional organization, dysfunction, and perspective.* Paper presented at the Mayo Clinic Proceedings.

Blackledge, J. T. (2003). An introduction to relational frame theory: Basics and applications. *The Behavior Analyst Today, 3*(4), 421.

Bohlen, L., Shaw, R., Cerritelli, F., & Esteves, J. E. (2021). Osteopathy and Mental Health: An Embodied, Predictive, and Interoceptive Framework. *Frontiers in psychology*, 4989.

Borst, A., & Theunissen, F. E. (1999). Information theory and neural coding. *Nature neuroscience, 2*(11), 947-957.

Buijs, R. M., & Van Eden, C. G. (2000). The integration of stress by the hypothalamus, amygdala and prefrontal cortex: balance between the autonomic nervous system and the neuroendocrine system. In *Progress in brain research* (Vol. 126, pp. 117-132): Elsevier.

Carver, C. S., & Scheier, M. F. (2002). Control processes and self-organization as complementary principles underlying behavior. *Personality and social psychology review, 6*(4), 304-315.

Charan, A. R., Gharibzadeh, S., & Firouzabadi, S. M. (2021). Realism is almost true: A critique of the interface theory of perception. *arXiv preprint arXiv:2111.03864*.

Cisek, P. (2007). Cortical mechanisms of action selection: the affordance competition hypothesis. *Philosophical Transactions of the Royal Society B: Biological Sciences, 362*(1485), 1585-1599.

Cisek, P., & Kalaska, J. F. (2010). Neural mechanisms for interacting with a world full of action choices. *Annual review of neuroscience, 33*, 269-298.

Craig. (2002). How do you feel? Interoception: the sense of the physiological condition of the body. *Nature reviews neuroscience, 3*(8), 655.

Craig. (2003). Interoception: the sense of the physiological condition of the body. *Current opinion in neurobiology, 13*(4), 500-505.

Craig. (2014). *How do you feel?: an interoceptive moment with your neurobiological self*.

Crauel, H., & Flandoli, F. (1994). Attractors for random dynamical systems. *Probability Theory and Related Fields, 100*(3), 365-393.

Critchley, H. (2003). Emotion and its disorders: Imaging in clinical neuroscience. *British Medical Bulletin, 65*(1), 35-47.

Damasio. (2003). Mental self: The person within. *Nature, 423*(6937), 227-227.

Damasio, & Carvalho. (2013). The nature of feelings: evolutionary and neurobiological origins. *Nature reviews neuroscience, 14*(2), 143.

Depew, D. J., & Weber, B. H. (1996). Darwinism evolving: Systems dynamics and the genealogy of natural selection. *British Journal for the Philosophy of Science, 47*(4).

El Kaabouchi, A., & Wang, Q. A. (2015). *Least action principle and stochastic motion: a generic derivation of path probability.* Paper presented at the Journal of Physics: Conference Series.
England, J. L. (2015). Dissipative adaptation in driven self-assembly. *Nature nanotechnology, 10*(11), 919-923.
Evans, D. J., & Searles, D. J. (2002). The fluctuation theorem. *Advances in Physics, 51*(7), 1529-1585.
Friston. (2009). The free-energy principle: a rough guide to the brain? *Trends in cognitive sciences, 13*(7), 293-301.
Friston. (2010). The free-energy principle: a unified brain theory? *Nature Reviews Neuroscience, 11*(2), 127-138.
Friston. (2013). Active inference and free energy. *Behavioral and brain sciences, 36*(3), 212.
Friston. (2019). A free energy principle for a particular physics. *arXiv preprint arXiv:1906.10184*.
Friston, Kilner, J., & Harrison, L. (2006). A free energy principle for the brain. *Journal of physiology-Paris, 100*(1-3), 70-87.
Friston, Parr, T., & de Vries, B. (2017). The graphical brain: belief propagation and active inference. *Network Neuroscience, 1*(4), 381-414.
Georgiev, G., & Georgiev, I. (2002). The least action and the metric of an organized system. *Open systems & information dynamics, 9*(4), 371-380.
Georgiev, G. Y., & Chatterjee, A. (2016). The road to a measurable quantitative understanding of self-organization and evolution. In *Evolution and Transitions in Complexity* (pp. 223-230): Springer.
Georgiev, G. Y., Chatterjee, A., & Iannacchione, G. (2017). Exponential self-organization and moore's law: Measures and mechanisms. *Complexity, 2017*.
Georgiev, G. Y., Henry, K., Bates, T., Gombos, E., Casey, A., Daly, M., . . . Lee, H. (2015). Mechanism of organization increase in complex systems. *Complexity, 21*(2), 18-28.
Gibson, J. J. (2014). *The ecological approach to visual perception: classic edition*: Psychology Press.
Gödel, K. (1931). Über formal unentscheidbare Sätze der Principia Mathematica und verwandter Systeme I. *Monatshefte für mathematik und physik, 38*(1), 173-198.
Grassberger, P., & Procaccia, I. (1983). Characterization of strange attractors. *Physical review letters, 50*(5), 346.
Hayes, S. C., Strosahl, K. D., Bunting, K., Twohig, M., & Wilson, K. G. (2004). What is acceptance and commitment therapy? In *A practical guide to acceptance and commitment therapy* (pp. 3-29): Springer.
Hayes, S. C., Strosahl, K. D., & Wilson, K. G. (1999). *Acceptance and commitment therapy*: Guilford press New York.
Hayes, S. C., Strosahl, K. D., & Wilson, K. G. (2011). *Acceptance and commitment therapy: The process and practice of mindful change*: Guilford press.
Hipolito, I. (2019). A simple theory of every 'thing'. *Physics of life reviews, 31*, 79-85.
Hirsh, J. B., Mar, R. A., & Peterson, J. B. (2012). Psychological entropy: a framework for understanding uncertainty-related anxiety. *Psychological review, 119*(2), 304.
Hofmann, S. G., Hayes, S. C., & Lorscheid, D. N. (2021). *Learning process-based therapy: A skills training manual for targeting the core processes of psychological change in clinical practice*: New Harbinger Publications.
Hofstadter, D. R. (1979). *Gödel, escher, bach*: Basic books New York.
Hofstadter, D. R. (2007). *I am a strange loop*: Basic books.
Hohwy, J. (2020). New directions in predictive processing. *Mind & language, 35*(2), 209-223.
Hollis, G., Kloos, H., & Van Orden, G. C. (2009). Origins of order in cognitive activity.
Kauffman, S. A. (1993). *The origins of order: Self-organization and selection in evolution*: Oxford University Press, USA.
Kelso, J. S. (1995). *Dynamic patterns: The self-organization of brain and behavior*: MIT press.
Kirchhoff. (2018). Hierarchical Markov blankets and adaptive active inference. *Physics of life reviews, 24*.

Koenderink, J. (2014). The all seeing eye? In (Vol. 43, pp. 1-6): SAGE Publications Sage UK: London, England.
LeDoux. (2000). Cognitive–emotional interactions: Listen to the brain.
LeDoux. (2020). Thoughtful feelings. *Current Biology, 30*(11), R619-R623.
LeDoux, & Brown, R. (2017). A higher-order theory of emotional consciousness. *Proceedings of the National Academy of Sciences, 114*(10), E2016-E2025.
Liapunov, A. M. (2016). *Stability of motion*: Elsevier.
Limanowski, J., & Friston, K. (2020). Attenuating oneself. *Philosophy and the Mind Sciences, 1*(I), 1-16.
Lundy Jr, R. F., & Norgren, R. (2004). Activity in the hypothalamus, amygdala, and cortex generates bilateral and convergent modulation of pontine gustatory neurons. *Journal of neurophysiology, 91*(3), 1143-1157.
Lyapunov, A. M. (1992). The general problem of the stability of motion. *International journal of control, 55*(3), 531-534.
Mark, J. T., Marion, B. B., & Hoffman, D. D. (2010). Natural selection and veridical perceptions. *Journal of Theoretical Biology, 266*(4), 504-515.
McEwen, B. S., Nasca, C., & Gray, J. D. (2016). Stress effects on neuronal structure: hippocampus, amygdala, and prefrontal cortex. *Neuropsychopharmacology, 41*(1), 3-23.
Murphy, F. C., Nimmo-Smith, I., & Lawrence, A. D. (2003). Functional neuroanatomy of emotions: a meta-analysis. *Cognitive, affective, & behavioral neuroscience, 3*(3), 207-233.
Nemenman, I., Bialek, W., & Van Steveninck, R. D. R. (2004). Entropy and information in neural spike trains: Progress on the sampling problem. *Physical review E, 69*(5), 056111.
Paninski, L. (2003). Estimation of entropy and mutual information. *Neural computation, 15*(6), 1191-1253.
Panneton, W. M. (1991). Primary afferent projections from the upper respiratory tract in the muskrat. *Journal of comparative neurology, 308*(1), 51-65.
Pereda, E., Quiroga, R. Q., & Bhattacharya, J. (2005). Nonlinear multivariate analysis of neurophysiological signals. *Progress in neurobiology, 77*(1-2), 1-37.
Phillips, R., & LeDoux, J. (1992). Differential contribution of amygdala and hippocampus to cued and contextual fear conditioning. *Behavioral neuroscience, 106*(2), 274.
Pinna, T., & Edwards, D. J. (2020). A Systematic Review of Associations Between Interoception, Vagal Tone, and Emotional Regulation: Potential Applications for Mental Health, Wellbeing, Psychological Flexibility, and Chronic Conditions.
Pollatos, O., Gramann, K., & Schandry, R. (2007). Neural systems connecting interoceptive awareness and feelings. *Human brain mapping, 28*(1), 9-18.
Porges, S. W. (2003). The polyvagal theory: Phylogenetic contributions to social behavior. *Physiology & behavior, 79*(3), 503-513.
Porges, S. W. (2007). A phylogenetic journey through the vague and ambiguous Xth cranial nerve: A commentary on contemporary heart rate variability research. *Biological psychology, 74*(2), 301-307.
Porges, S. W. (2018). Polyvagal theory: A primer. *Clinical applications of polyvagal theory: The emergence of polyvagal-informed therapies*, 50-69.
Prigogine, I. (1978). Time, structure, and fluctuations. *Science, 201*(4358), 777-785.
Prigogine, I., & Stengers, I. (1997). *The end of certainty: Time chaos, and the new laws of nature* New York, NY: Free Press.
Rabinak, C. A., Angstadt, M., Welsh, R. C., Kennedy, A., Lyubkin, M., Martis, B., & Phan, K. L. (2011). Altered amygdala resting-state functional connectivity in post-traumatic stress disorder. *Frontiers in psychiatry, 2*, 62.
Ramstead, Kirchhoff, M. D., & Friston, K. J. (2020). A tale of two densities: Active inference is enactive inference. *Adaptive Behavior, 28*(4), 225-239.
Schrödinger, E. (1942). *What is life?* : Cambridge University Press.

Schrödinger, E. (1944). What is life? The physical aspect of the living cell. *What is life? The physical aspect of the living cell.*

Shannon, C. E. (1948). A mathematical theory of communication. *The Bell system technical journal, 27*(3), 379-423.

Shin, L. M., Rauch, S. L., & Pitman, R. K. (2006). Amygdala, medial prefrontal cortex, and hippocampal function in PTSD. *Annals of the New York Academy of Sciences, 1071*(1), 67-79.

Stephen, D. G., Boncoddo, R. A., Magnuson, J. S., & Dixon, J. A. (2009). The dynamics of insight: Mathematical discovery as a phase transition. *Memory & Cognition, 37*(8), 1132-1149.

Stephen, D. G., Dixon, J. A., & Isenhower, R. W. (2009). Dynamics of representational change: entropy, action, and cognition. *Journal of Experimental Psychology: Human Perception and Performance, 35*(6), 1811.

Stevens, F. L., Hurley, R. A., & Taber, K. H. (2011). Anterior cingulate cortex: unique role in cognition and emotion. *The Journal of neuropsychiatry and clinical neurosciences, 23*(2), 121-125.

Strigo, I. A., & Craig, A. D. (2016). Interoception, homeostatic emotions and sympathovagal balance. *Philosophical Transactions of the Royal Society B: Biological Sciences, 371*(1708), 20160010.

Strong, S. P., Koberle, R., Van Steveninck, R. R. D. R., & Bialek, W. (1998). Entropy and information in neural spike trains. *Physical review letters, 80*(1), 197.

Sutherland, R., & McDonald, R. (1990). Hippocampus, amygdala, and memory deficits in rats. *Behavioural brain research, 37*(1), 57-79.

Thayer, & Friedman, B. H. (2002). Stop that! Inhibition, sensitization, and their neurovisceral concomitants. *Scandinavian Journal of Psychology, 43*(2), 123-130.

Thayer, & Friedman, B. H. (2004). 15 A Neurovisceral Integration Model of Health Disparities in Aging.

Thayer, & Lane. (2000). A model of neurovisceral integration in emotion regulation and dysregulation. *Journal of affective disorders, 61*(3), 201-216.

Tononi, G., Sporns, O., & Edelman, G. M. (1994). A measure for brain complexity: relating functional segregation and integration in the nervous system. *Proceedings of the National Academy of Sciences, 91*(11), 5033-5037.

Vallacher, R. R., Read, S. J., & Nowak, A. (2002). The dynamical perspective in personality and social psychology. *Personality and social psychology review, 6*(4), 264-273.

Warren, W. H. (2006). The dynamics of perception and action. *Psychological review, 113*(2), 358.

White, T. P., Joseph, V., Francis, S. T., & Liddle, P. F. (2010). Aberrant salience network (bilateral insula and anterior cingulate cortex) connectivity during information processing in schizophrenia. *Schizophrenia research, 123*(2-3), 105-115.

Zettle, R. D., Hayes, S. C., Barnes-Holmes, D., & Biglan, A. (2016). *The Wiley handbook of contextual behavioral science*: John Wiley & Sons.

Zhang, J., & Patel, V. L. (2006). Distributed cognition, representation, and affordance. *Pragmatics & Cognition, 14*(2), 333-341.
